# The *Campylobacter jejuni* Oxidative Stress Regulator RrpB Is Associated with a Genomic Hypervariable Region and Altered Oxidative Stress Resistance

**DOI:** 10.3389/fmicb.2016.02117

**Published:** 2016-12-26

**Authors:** Ozan Gundogdu, Daiani T. da Silva, Banaz Mohammad, Abdi Elmi, Brendan W. Wren, Arnoud H. M. van Vliet, Nick Dorrell

**Affiliations:** ^1^Faculty of Infectious and Tropical Diseases, London School of Hygiene and Tropical MedicineLondon, UK; ^2^School of Veterinary Medicine, Faculty of Health and Medical Sciences, University of SurreyGuildford, UK

**Keywords:** *Campylobacter jejuni* oxidative stress response regulation, aerobic stress response regulation, transcription factors, restriction modification system

## Abstract

*Campylobacter jejuni* is the leading cause of bacterial foodborne diarrhoeal disease worldwide. Despite the microaerophilic nature of the bacterium, *C. jejuni* can survive the atmospheric oxygen conditions in the environment. Bacteria that can survive either within a host or in the environment like *C. jejuni* require variable responses to survive the stresses associated with exposure to different levels of reactive oxygen species. The MarR-type transcriptional regulators RrpA and RrpB have recently been shown to play a role in controlling both the *C. jejuni* oxidative and aerobic stress responses. Analysis of 3,746 *C. jejuni* and 486 *C. coli* genome sequences showed that whilst *rrpA* is present in over 99% of *C. jejuni* strains, the presence of *rrpB* is restricted and appears to correlate with specific MLST clonal complexes (predominantly ST-21 and ST-61). *C. coli* strains in contrast lack both *rrpA* and *rrpB*. In *C. jejuni rrpB*^+^ strains, the *rrpB* gene is located within a variable genomic region containing the IF subtype of the type I Restriction-Modification (*hsd*) system, whilst this variable genomic region in *C. jejuni rrpB*^-^ strains contains the IAB subtype *hsd* system and not the *rrpB* gene. *C. jejuni rrpB*^-^ strains exhibit greater resistance to peroxide and aerobic stress than *C. jejuni rrpB*^+^ strains. Inactivation of *rrpA* resulted in increased sensitivity to peroxide stress in *rrpB*^+^ strains, but not in *rrpB*^-^ strains. Mutation of *rrpA* resulted in reduced killing of *Galleria mellonella* larvae and enhanced biofilm formation independent of *rrpB* status. The oxidative and aerobic stress responses of *rrpB*^-^ and *rrpB*^+^ strains suggest adaptation of *C. jejuni* within different hosts and niches that can be linked to specific MLST clonal complexes.

## Introduction

*Campylobacter jejuni* is the leading cause of bacterial foodborne diarrhoeal disease worldwide with an estimated 400 million human infections occurring each year (Ruiz-Palacios, 2007). The predominance of *C. jejuni* can be attributed to the ability to survive in the environment as well as within avian and mammalian hosts despite the microaerophilic nature of this bacterium (Byrne et al., 2007). *C. jejuni* has evolved specific adaptation mechanisms to survive under atmospheric oxygen conditions (Kim et al., 2015). In addition to aerobic stress such as the exposure to increased levels of oxygen under atmospheric conditions, *C. jejuni* can also encounter stress conditions within the host, specifically oxidative stress in the form of reactive oxygen species (ROS) during *in vivo* survival (Fang, 2004; Palyada et al., 2009). ROS is a collective term that describes the chemical species generated upon incomplete reduction of oxygen (Imlay, 2003) with examples including the superoxide anion (O_2_^-^), hydrogen peroxide (H_2_O_2_) and the hydroxyl radical (^•^OH) (D’Autreaux and Toledano, 2007). The accumulation of ROS in the bacterial cytoplasm and periplasm leads to damage of nucleic acids, proteins and membrane structures (Atack and Kelly, 2009).

Bacteria that can survive either within a host or in the environment like *C. jejuni* require variable responses to survive the stresses associated with exposure to different levels of ROS (Kim et al., 2015). Therefore it is not surprising that *C. jejuni* contains a number of regulatory proteins involved in the oxidative stress response such as PerR (Handley et al., 2015), Fur (van Vliet et al., 2000), and CosR (Hwang et al., 2011). The *C. jejuni* NCTC 11168 genome also contains two MarR-type transcriptional regulators that have previously been designated as RrpA and RrpB (Gundogdu et al., 2011, 2015). Using *C. jejuni* 11168H [a hypermotile derivative of the original sequenced strain NCTC 11168 that shows higher levels of cecal colonization in a chick colonization model (Karlyshev et al., 2002; Jones et al., 2004)] we have shown that both RrpA and RrpB play a role in oxidative and aerobic stress responses with auto-regulatory activity typical of MarR-type transcriptional regulators (Gundogdu et al., 2011, 2015). In addition, RrpA has also been shown to bind upstream of *katA* suggesting that RrpA directly influences the expression of catalase (KatA). Both 11168H *rrpA* and *rrpB* mutants exhibited reduced KatA activity. However, a 11168H *rrpAB* double mutant exhibited higher levels of resistance to hydrogen peroxide oxidative stress, but similar levels of KatA activity compared to the wild-type strain. Neither the 11168H *rrpA* mutant nor the 11168H *rrpB* mutant exhibited any significant difference in sensitivity to either cumene hydroperoxide or menadione oxidative stresses, but both mutants exhibited reduced cytotoxicity in the *Galleria mellonella* model of infection and enhanced biofilm formation. However, the 11168H *rrpAB* double mutant exhibited wild-type levels of both cytotoxicity in the *G. mellonella* model of infection and biofilm formation. Together these data indicate a role for both RrpA and RrpB in the *C. jejuni* oxidative and aerobic stress responses, enhancing bacterial survival both within a host and in the environment, but also prompted further investigations in order to understand the specific roles of RrpA and RrpB.

Traditional typing methods have failed to identify *C. jejuni* strains from different sources that cause disease in humans (Champion et al., 2005). Human infections are largely attributed to undercooking of poultry products or poor food hygiene practices involving handling of such produce (Sheppard et al., 2009). *C. jejuni* can survive in many different niches with the organism isolated from avian, animal, human, and environmental sources (Young et al., 2007). However, whole genome phylogenetic analysis of *C. jejuni* strains using microarrays has identified different clades and subclades linked to the source of the isolate (Champion et al., 2005; Stabler et al., 2013). Previously we identified differences in the distribution of *rrpA* and *rrpB* regulators amongst 111 *C. jejuni* strains with *rrpA* present in over 95% and *rrpB* in approximately only 50% of these strains (Gundogdu et al., 2011). A more recent analysis of 270 *C. jejuni* strains identified nine clusters (C1–C9) based on genotype and multilocus sequence typing (MLST) data and indicated that clusters C1–C6 were dominated by livestock-associated clonal complexes, whilst clusters C7–C9 contained the majority of the water and wildlife associated clonal complexes (Stabler et al., 2013). Using the original data from this study, *rrpA* was identified in 129/133 (96.99%) strains within the C1–C6 subclades and in 130/137 (94.89%) strains within the C7–C9 subclades. In contrast *rrpB* was identified in 102/133 (76.67%) strains within the C1–C6 subclades and only in 19/137 (13.87%) strains within the C7–C9 subclades. In total *rrpA* was identified in 259/270 (95.92%) strains whilst *rrpB* was identified in only in 121/270 (44.81%) strains (Supplementary Figure 1; Supplementary Table 1).

The discovery of the varied distribution for *rrpA* and *rrpB* has led us to further explore the potential reasons as to why certain strains have one or both regulators. To try and understand the reasons for this, we have investigated the presence or absence of *rrpA* and *rrpB* in 4,232 *C. jejuni* and *C. coli* genome sequences. Analysis of 4,232 *Campylobacter* genomes showed that whilst *rrpA* is present in over 99% of *C. jejuni* strains, the presence of *rrpB* is restricted and appears to correlate with livestock-associated MLST clonal complexes. Further analysis showed that the presence of *rrpB* is linked to a hypervariable region containing the IF subtype of the type I Restriction-Modification (*hsd*) system, whereas *rrpB*^-^ strains contain the IAB subtype *hsd* system. Further investigation of the phenotypes of different *C. jejuni rrpB*^-^ and *rrpB*^+^ strains identified a link between the presence of the MarR-type transcriptional regulator RrpB with the ability of *C. jejuni* to adapt and survive in different environmental niches. The oxidative and aerobic stress response of *rrpB*^-^ and *rrpB*+ strains suggests adaptation of *C. jejuni* within different hosts and niches that can be linked with specific MLST clonal complexes.

## Materials and Methods

### Comparative Genomics of *rrpA* and *rrpB* within *Campylobacter* Genomes

A total of 4,232 complete and draft *Campylobacter* genome sequences (3,746 *C. jejuni* and 486 *C. coli*) were obtained from public collections (Jolley and Maiden, 2010; Cody et al., 2013) and are listed in Supplementary Table 2 with accession numbers/pubMLST IDs and assembly status. These genomes were previously used for identification of DNase-genes (Brown et al., 2015), CRISPR repeats and *cas* genes (Pearson et al., 2015) and the fucose utilization operon (Dwivedi et al., 2016). Genomes included were between 1.5 and 2.0 Mbp and had at least 5 of the 7 MLST alleles identifiable using BLAST. The genomes were phylogenetically clustered using FFPry feature frequency profiling with a word length of 18 (Van Vliet and Kusters, 2015) and further analyzed by MLST (Pearson et al., 2015). Genomes were provisionally annotated using Prokka (Seemann, 2014) and searched for the presence of the predicted RrpA and RrpB proteins using BLASTP (Supplementary Table 2). Presence of the corresponding genes was also assessed at the DNA level using the MIST program (Kruczkiewicz et al., 2013) and the BLAST+ (v2.28) suite. Both DNA and amino acid comparisons were done using the *rrpA* genes/RrpA proteins from *C. jejuni* NCTC 11168, *C. jejuni* 81116, *C. jejuni* 414, and *C. coli* 76639, and the *rrpB* genes/RrpB proteins of *C. jejuni* NCTC 11168 and *C. coli* 2544. Conservation of flanking genes was assessed using the output from the comparative genomics software package Roary (Page et al., 2015) and the provisional Prokka annotation of the 4,232 *C. jejuni* and *C. coli* genome sequences (Supplementary Tables 2 and 3).

### Bacterial Strains and Growth Conditions

*Campylobacter jejuni* strains (**Table 1**) were grown at 37°C in a microaerobic chamber (Don Whitley Scientific, United Kingdom) containing 85% N_2_, 10% CO_2_ and 5% O_2_ either on blood agar (BA) plates containing Columbia agar base (Oxoid, United Kingdom) supplemented with 7% (v/v) horse blood (TCS Microbiology, United Kingdom) and *Campylobacter* Selective Supplement (Oxoid) or in Brucella broth (Oxoid) with shaking at 75 rpm. *C. jejuni* strains were grown on BA plates for 24 h prior to use in all assays unless otherwise stated. *Escherichia coli* XL-2 Blue MRF’ competent cells (Stratagene, United Kingdom) were used for cloning experiments and were grown at 37°C in aerobic conditions either on Luria-Bertani (LB) agar plates (Oxoid) or in LB broth (Oxoid) with shaking at 200 rpm. Antibiotics were added at the following concentrations; ampicillin (100 μg/ml), kanamycin (50 μg/ml), and chloramphenicol (50 μg/ml for *E. coli* studies or 10 μg/ml for *C. jejuni* studies). All reagents were obtained from Fisher Scientific (United Kingdom) unless otherwise stated.

**Table 1:** *Campylobacter jejuni* strains used in this study.

Strain	Description	Reference
11168H	A hypermotile derivative of the original sequence strain NCTC 11168 that shows higher levels of caecal colonization in a chick colonization model. C3 clade. MLST clonal complex ST-21.	Karlyshev et al., 2002; Jones et al., 2004
81-176	Highly virulent and widely studied laboratory strain of *C. jejuni*. MLST clonal complex ST-42.	Korlath et al., 1985
81116	Genetically stable strain which remains infective in avian models. C9ii clade. MLST clonal complex ST-283.	Wassenaar et al., 1991
M1	M1 (laboratory designation 99/308) is a rarely documented case of direct transmission of *C. jejuni* from chicken to a person, resulting in enteritis. C9ii clade. MLST clonal complex ST-45.	Friis et al., 2010
11168H *rrpB* mutant	Isogenic 11168H *rrpB* mutant with insertion of a 1.4 kb Kan^R^ cassette	Gundogdu et al., 2011
11168H *rrpB* complement	*rrpB* complement constructed by the insertion of the *rrpB* CDS into the *Cj0233* pseudogene in the 11168H *rrpB* mutant (pDENNIS complementation vector used)	Gundogdu et al., 2011
81-176 *rrpB* mutant	Isogenic 81-176 *rrpB* mutant with insertion of a 1.4 kb Kan^R^ cassette	This study
81-176 *rrpB* complement	*rrpB* complement constructed by the insertion of the *rrpB* CDS into a rRNA gene in the 81-176 *rrpB* mutant (pRRC complementation vector used)	This study
11168H *rrpA* mutant	Isogenic 11168H *rrpA* mutant with insertion of a 1.4 kb Kan^R^ cassette.	Gundogdu et al., 2015
11168H *rrpA* complement	*rrpA* complement constructed by the insertion of the *rrpA* CDS into a rRNA gene in the 11168H *rrpA* mutant (pRRC complementation vector used).	Gundogdu et al., 2015
81-176 *rrpA* mutant	Isogenic 81-176 *Cj1546* mutant with insertion of a 1.4 kb Kan^R^ cassette	This study
81-176 *rrpA* complement	*rrpA* complement constructed by the insertion of the *rrpA* CDS into a rRNA gene in the 81-176 *rrpA* mutant (pRRC complementation vector used)	This study
81116 *rrpA* mutant	Isogenic 81116 *rrpA* mutant with insertion of a 1.4 kb Kan^R^ cassette	This study
81116 *rrpA* complement	*rrpA* complement constructed by the insertion of the *rrpA* CDS into a rRNA gene in the 81116 *rrpA* mutant (pRRC complementation vector used)	This study
M1 *rrpA* mutant	Isogenic M1 *rrpA* mutant with insertion of a 1.4 kb Kan^R^ cassette	This study
40917	Clinical bloody diarrhea isolate HS type 21 Water and wildlife associated strain. C9ii clade. MLST clonal complex ST-45.	*Campylobacter* Reference Lab, UK
12241	Ovine isolate HS type 50 Water and wildlife associated strain. C9ii clade. MLST clonal complex ST-206.	*Campylobacter* Reference Lab, UK
64555	Clinical bloody diarrhea isolate HS type 31 Water and wildlife associated strain. C8 clade.	*Campylobacter* Reference Lab, UK
47693	Isolated from chicken isolate HS type 27 Water and wildlife associated strain. C9ii clade. MLST clonal complex ST-45.	*Campylobacter* Reference Lab, UK
62914	Clinical vomiting isolate HS type – untypeable Water and wildlife associated strain. C7 clade.	*Campylobacter* Reference Lab, UK
44119	Clinical septicaemia isolate HS type – 18 Water and wildlife associated strain. C7 clade.	*Campylobacter* Reference Lab, UK
32787	Clinical asymptomatic isolate HS type – 18 Water and wildlife associated strain. C9i clade.	*Campylobacter* Reference Lab, UK
Hi80614	Human isolate Water and wildlife associated strain. C9i clade.	*Campylobacter* Reference Lab, UK
Hi41100305	Human isolate Water and wildlife associated strain. C9i clade.	*Campylobacter* Reference Lab, UK
31481	Clinical asymptomatic isolate HS type – 37 Water and wildlife associated strain. C8 clade.	*Campylobacter* Reference Lab, UK
47886	Clinical septicaemia isolate HS type – untypeable Livestock associated strain. C1 clade.	*Campylobacter* Reference Lab, UK
30280	Clinical diarrhea isolate HS type – 16 Livestock associated strain. C2 clade.	*Campylobacter* Reference Lab, UK
13713	Ox liver portion isolate HS type – 2 Livestock associated strain. C1 clade.	*Campylobacter* Reference Lab, UK
11973	Chicken isolate HS type – 2 Livestock associated strain. C1 clade.	*Campylobacter* Reference Lab, UK
G1	Clinical GBS isolate HS type – 1 Livestock associated strain. C5 clade. MLST clonal complex ST-21.	Guy’s Hospital, UK
Bovine27	Bovine isolate Livestock associated strain. C2 clade.	University of Bristol, UK
12912	Ox liver portion isolate HS type – 50 Livestock associated strain. C4 clade.	Campylobacter Reference Lab, UK
13040	Chicken isolate HS type – 50 Livestock associated strain. C6 clade.	Campylobacter Reference Lab, UK
40209	Chicken isolate HS type – 5 Livestock associated strain. C4 clade.	Campylobacter Reference Lab, UK
91B1	Chicken isolate Livestock associated strain. C5 clade.	Oxford University, UK
Hi41380304	Human isolate Livestock associated strain. C3 clade.	Campylobacter Reference Lab, UK
11168H *perR* mutant	Obtained from *Campylobacter* mutant bank http://crf.lshtm.ac.uk/wren\_mutants.htm	LSHTM mutant bank
11168H *sodB* mutant	Obtained from *Campylobacter* mutant bank http://crf.lshtm.ac.uk/wren\_mutants.htm	LSHTM mutant bank
11168H *ahpC* mutant	Obtained from *Campylobacter* mutant bank http://crf.lshtm.ac.uk/wren\_mutants.htm	LSHTM mutant bank
11168H *katA* mutant	Obtained from *Campylobacter* mutant bank http://crf.lshtm.ac.uk/wren\_mutants.htm	LSHTM mutant bank

### Construction of *C. jejuni* Mutants

*Campylobacter jejuni* mutants were constructed as described previously (Gundogdu et al., 2011). Briefly, genes or gene fragments were amplified from *C. jejuni* genomic DNA using the appropriate gene specific primers (**Table 2**). PCR products were ligated with pGEM-T Easy vector (Promega, United Kingdom) and then transformed into XL-2 Blue MRF’ cells. If required, inverse PCR mutagenesis (IPCRM) was performed to introduce a unique *Bgl*II site into the cloned gene. A kanamycin cassette (Kan^R^) was then ligated into the unique *Bgl*II site within the cloned gene (Trieu-Cuot et al., 1985; van Vliet et al., 1998). These constructs were electroporated into competent *C. jejuni* cells and putative clones were confirmed by PCR and sequencing as described previously (Gundogdu et al., 2011).

**Table 2:** Oligonucleotide primers used in this study.

Primer Name	Sequence
11168H and 81-176 *Cj155*6-F	ATCATTCTCTTTGTCCTAT
11168H and 81-176 *Cj1556*-R	TAAGATGGATTCTAAACTATTG
11168H Comp-*Cj1556*-F	CCCCCATGGATAAGGATTTATAATGAAAAAATATCATTCTCT
11168H Comp-*Cj1556*-R	CCCGCTAGCTTAAACGATATTTTTATAGCTAT
81-176 Comp-*Cj1556*-F	CCCTCTAGAATAAGGATTTATAATGAAAAAATATCATTCTCT
81-176 Comp-*Cj1556*-R	CCCTCTAGATTAAACGATATTTTTATAGCTAT
11168H and 81-176 *Cj154*6-F	TACTAGGATTTTCATGAG
11168H and 81-176 *Cj1546*-R	AGATGTTAAATCTCACTGCT
11168H and 81-176 *Cj1546* IPCR-F	GGGAGATCTCTCTTAAGGTATTGGTTA
11168H and 81-176 *Cj1546* IPCR-R	GGGAGATCTCGATGGTTTAATTATCAG
11168H and 81-176 Comp-*Cj1546*-F	CCCTCTAGACTAAAGGAATGTTAAATGACTAAAGAGAATTCTCCG
11168H and 81-176 Comp-*Cj1546*-R	CCCTCTAGATTAATTCAAGCATTTTTTCCC
11168H and 81-176 *Cj1546*-F checking primers	TTCTCCGTGCAATTTCG
11168H and 81-176 *Cj1546*-F checking primers	CCATTTGCTCATAGCTTGTAA
81116 *Cj154*6-F	ATGAGTTTGATCTACTTCG
81116 *Cj1546*-R	AGATATTAAATCTCACTGCT
81116 *Cj1546* IPCR-F	GGGAGATCTCTCTTAAGGTATTGGTTA
81116 *Cj1546* IPCR-R	GGGAGATCTTGATGGTTTAATTATCAG
81116 Comp-*Cj1546*-F	CCCTCTAGACTAAAGGAATGTTAAATGACTAAAGAGAATTCTCAG
81116 Comp-*Cj1546*-R	CCCTCTAGATTATCAAGCATTTTTTTCC
81116 *Cj1546*-F checking primers	ATGACTAAAGAGAATTCTC
81116 *Cj1546*-R checking primers	CCATTTGCACATAGCTTG
Kan^R^ forward-out	TGGGTTTCAAGCATTAGTCCATGCAAG
Kan^R^ reverse-out	GTGGTATGACATTGCCTTCTGCG
Cat^R^ forward-out	CGATTGATGATCGTTGTA
Cat^R^ reverse-out	TACAGCAGACTATACTG

### Oxidative Stress and Aerobic Growth Assays

Oxidative stress and aerobic growth assays were performed as described previously (Gundogdu et al., 2011, 2015). Briefly, bacterial cells were harvested into 1 ml PBS and diluted to an OD_600_ of 1. For oxidative stress assays, bacterial cells were exposed to H_2_O_2_ at final concentrations of 25, 50, or 100 mM for 15 min, menadione at a final concentration of 100 mM for 60 min and cumene hydroperoxide at 0.05% (w/v) for 15 min, all at 37°C under microaerobic conditions. Serial dilutions were prepared and 10 μl of the 10^-1^–10^-6^ dilutions spotted onto BA plates, incubated for 48 h and colonies counted. For growth curves, 10 ml Brucella broth was pre-incubated in a 30 ml flask at 37°C under microaerobic conditions for 24 h. Bacterial cells grown on BA plates for 24 h were used to inoculate pre-incubated Brucella broth at an OD_600_ of 0.1 and grown for up to 24 h at 37°C under microaerobic and aerobic conditions. OD_600_ readings were performed at selected time points. In addition bacterial colony forming units (CFUs) were assessed at time point 16 h under microaerobic and aerobic conditions.

### *Galleria mellonella* Infection Model

*Galleria mellonella* infection assays were performed as described previously (Champion et al., 2010; Gundogdu et al., 2015). Briefly, *G. mellonella* larvae (LiveFoods Direct, United Kingdom) were stored at 16°C on wood chips. Ten larvae for each experiment were infected with a 10 μl inoculum of a 24 h *C. jejuni* culture diluted to OD_600_ 0.1 by micro-injection (Hamilton, Switzerland) in the right foremost leg, giving an infectious dose of approximately 10^6^ CFU (Champion et al., 2010). Controls were injection with PBS and no injection. Larvae were incubated at 37°C with survival recorded at 24 h.

### Biofilm Assays

Biofilm assays were performed as described previously (Gundogdu et al., 2015). Briefly, bacterial cells were harvested into Mueller Hinton broth, then inoculated to an OD_600_ of 0.1 into 10 ml Mueller Hinton broth pre-incubated in a 25 ml flask at 37°C under microaerobic conditions for 24 h prior to inoculation, then grown for 5 h at 37°C under microaerobic conditions with shaking at 75 rpm. The OD_600_ was readjusted to 0.1, then 1 ml of culture was added to a 24 well polystyrene plate (Corning, U.S.A) and incubated at 37°C under either aerobic or microaerobic conditions stationary for 72 h. The wells were washed twice with PBS, dried for 20 min at 37°C followed by addition of 1% (w/v) crystal violet (Sigma–Aldrich) for 15 min. The wells were washed three times with PBS, then destained with 10% (v/v) acetic acid / 30% (v/v) methanol. Absorbance (*A*_595_) was measured using a SpectraMax M3 microplate reader (Molecular Devices, USA).

### Statistical Analyses

The data is presented as mean + SD. All experiments were performed with at least three biological replicates. Each biological replicate was performed in three technical replicates. Statistical analyses were performed using Prism software (GraphPad Software). All statistical analyses were performed comparing two data sets directly assuming a normal distribution using a two-way student’s *t*-test (^∗^ = *p* < 0.05, ^∗∗^ = *p* < 0.01, ^∗∗∗^ = *p* < 0.001).

## Results

### Presence of *rrpA* and *rrpB* in a Collection of 4,232 *C. jejuni* and *C. coli*

**FIGURE 1:**
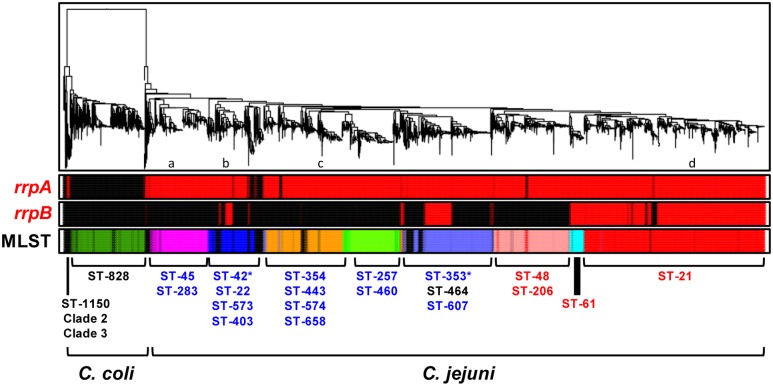
**Prevalence of the *rrpA* and *rrpB* genes among 3,746 *Campylobacter jejuni* and 486 *C. coli* genomes**. Genomes were phylogenetically clustered using FFPry feature frequency profiling with *L* = 18 (Van Vliet and Kusters, 2015). The first two rows labeled ‘*rrpA’* and ‘*rrpB’* indicate genomes possessing the respective genes, which are shown in red, while those lacking the pathway are shown in black. The third bar shows the primary combinations of MLST clonal complexes for *C. jejuni* and *C. coli*, with red-labeled clonal complexes representing livestock-associated lineages, blue-labeled clonal complexes representing water and wildlife-associated lineages (Stabler et al., 2013), with the exception of the ST-61 clonal complex, which has been described as cattle (livestock)-associated (Kwan et al., 2008; Rotariu et al., 2009). The asterisks at ST-42 and ST-353 indicate that these clonal complexes may have a proportion of livestock-associated isolates. The association of some clonal complexes such as ST-464 was not reported previously and these are in black font. The lowercase letters indicate the approximate position of reference strains 81116 and M1 (a), 81-176 (b), RM1221 (c) and NCTC 11168 (d).

To assess the distribution of *rrpA* and *rrpB* in *C. jejuni* and *C. coli* we utilized a collection of 4,232 *Campylobacter* genome sequences (3,746 *C. jejuni* and 486 *C. coli*) from public databases (Cody et al., 2013; Brown et al., 2015), which were phylogenetically clustered using FFPry feature frequency profiling (Van Vliet and Kusters, 2015; Dwivedi et al., 2016) and further analyzed for MLST sequence type and clonal complex (Pearson et al., 2015). The vast majority of *C. jejuni* strains contain *rrpA* whilst the presence of *rrpB* is more restricted (**Figure 1**; Supplementary Table 2 – Sheet 2). The presence or absence of *rrpB* appears to correlate with MLST clonal complex, especially ST-21 and ST-61. Most *C. coli* strains contain neither *rrpA* nor *rrpB*, with only 12 *C. coli* genomes encoding an RrpA ortholog and only a single *C. coli* genome encoding an RrpB ortholog (Supplementary Table 2). Variation in RrpA and RrpB was observed with isolates from *C. jejuni* MSLT clonal complex ST-607 having a shorter RrpA protein lacking the N-terminal 27 amino acids, while a proportion of *C. jejuni* ST-353 isolates have a RrpB protein either lacking the N-terminal 29 amino acids, the C-terminal 29 amino acids or a combination of 29 N-terminal amino acids and 9 C-terminal amino acids (Supplementary Figure 2). There was no apparent link between the truncated versions and source of the isolates (Supplementary Table 2), suggesting these are different forms of RrpA and RrpB circulating within clonal complexes.

**FIGURE 2:**
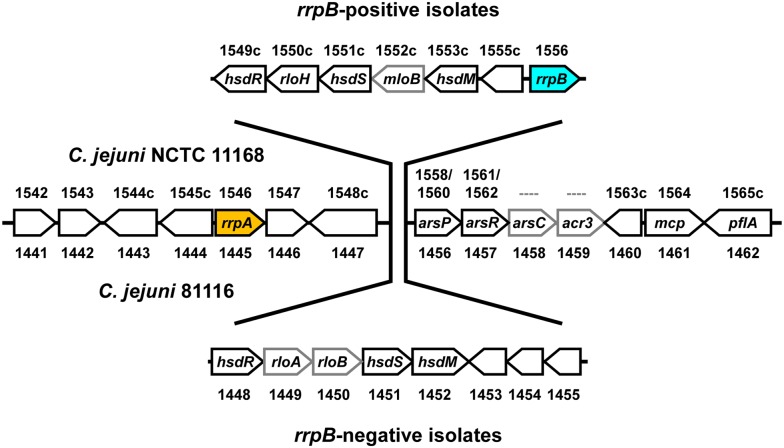
**NCTC 11168 type (*rrpB*^+^ isolate) and 81116 type (*rrpB*^-^ isolate) differ in a hypervariable region containing a type I Restriction-Modification (*hsd*) system**. The genetic structure around *rrpA* is conserved in *C. jejuni* strains, however, *rrpB* appears to be part of a transferable hypervariable region linked with variation in the type I Restriction-Modification (*hsd*) system, suggesting a mechanism for the variable distribution of *rrpB*. The figure shows the *C jejuni* NCTC 11168 (*rrpB*^+^) and 81116 (*rrpB*^-^) genes and gene numbers (Supplementary Table 3).

Further analysis of the *C. jejuni* and *C. coli* genome sequences investigated the distribution of genes flanking *rrpA* and *rrpB* and identified two conserved flanking regions with a more variable central region (**Figure 2**). The upstream flanking region contains *rrpA* whilst the downstream flanking region contains an arsenic resistance operon, an MCP-gene and a paralyzed flagella gene *pflA*. The hypervariable central region contains a type I Restriction-Modification (*hsd*) system. The NCTC 11168 version of this hypervariable central region contains *rrpB* whilst the 81116 version does not (Supplementary Table 3). The 81116 version of this hypervariable central region is representative of all the other *C. jejuni* ST strains analyzed in this study. Analysis of the *C. coli* genome sequences (which have primarily a structure very similar to 81116) has versions mostly without *rrpA* and *rrpB*.

### *Campylobacter jejuni rrpB^-^* Wild-Type Strains Exhibit Increased Resistance to Oxidative Stress Compared to *C. jejuni rrpB*^+^ Wild-Type Strains

**FIGURE 3:**
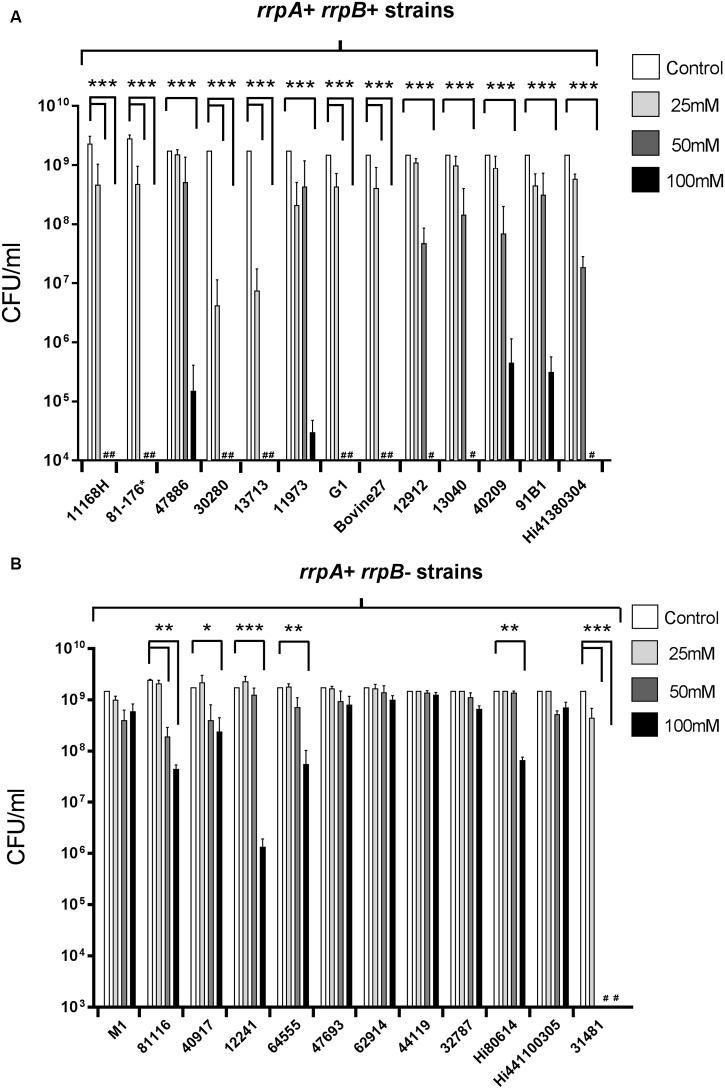
**Effect of oxidative stress on the survival of *C. jejuni rrpB*^+^**(A)** and *rrpB*^-^**(B)** wild-type strains**. *rrpB*^+^ strains are 11168H, 81-176, 47886, 30280, 13713, 11973, G1, Bovine27, 12912, 13040, 40209, 91B1, and Hi41380304. *rrpB*^-^ strains are M1, 81116, 40917, 12241, 64555, 47693, 62914, 44119, 32787, Hi80614, Hi441100305, and 31481. These strains were selected from the Stabler et al. (2013) study (**Table 1**). *C. jejuni* strains were incubated with 25 mM, 50 or 100 mM H_2_O_2_ for 15 min at 37°C under microaerobic conditions. ^∗^For 81-176 denotes that this strain was not included in Stabler et al. (2013), but was selected here as a common laboratory strain. Bacterial survival was subsequently assessed. # Symbol indicates lack of growth. Asterisks denote a statistically significant difference (^∗^ = *p* < 0.05, ^∗∗^ = *p* < 0.01, ^∗∗∗^ = *p* < 0.001) between strains.

Characterization of the role of RrpA and RrpB in the *C. jejuni* 11168H wild-type strain has shown that both *rrpA* and *rrpB* mutants exhibit increased sensitivity to H_2_O_2_ when compared to the wild-type strain (Gundogdu et al., 2011, 2015). Based on the observed variation of *rrpA* and *rrpB* amongst *C. jejuni* isolates (Gundogdu et al., 2011), we utilized the Stabler et al. (2013) study to select 25 wild-type strains (**Table 1**) (13 *rrpB*^+^ and 12 *rrpB*^-^ strains) for testing sensitivity toward H_2_O_2_ (**Figure 3**). *rrpB*^-^ strains (**Figure 3B**) displayed significantly greater resistance to H_2_O_2_ when compared to *rrpB*^+^ strains (**Figure 3A**). The variation in the presence of *rrpA* and *rrpB* identified from the microarray data (Champion et al., 2005) for these 25 strains was also confirmed using PCR (data not shown).

### Mutation of *rrpA* Results in Increased Sensitivity to Peroxide Stress in Strains 11168H and 81-176 (*rrpB*^+^), But Not in Strains 81116 and M1 (*rrpB*^-^)

**FIGURE 4:**
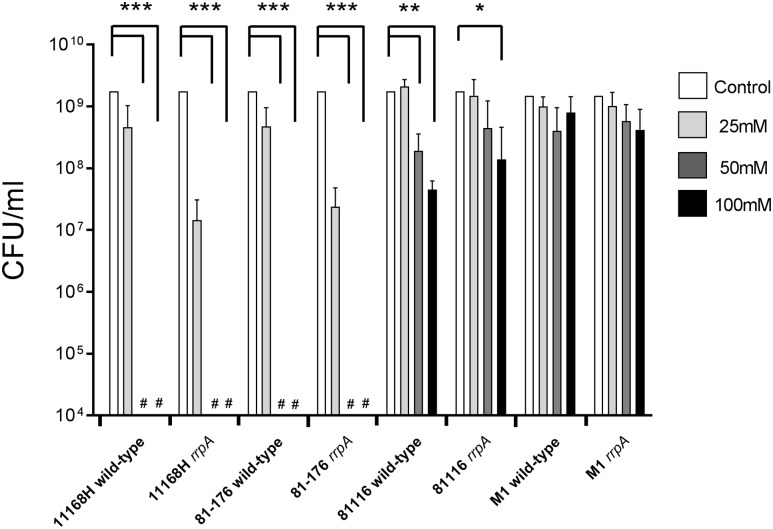
**Effect of oxidative stress on the survival of *C. jejuni* wild-type strains 11168H, 81-176, 81116, and M1 and respective *rrpA* mutants**. *C. jejuni* strains were incubated with 25, 50, or 100 mM H_2_O_2_ for 15 min at 37°C under microaerobic conditions. Bacterial survival was subsequently assessed. # Symbol indicates lack of growth. Asterisks denote a statistically significant difference (^∗^ = *p* < 0.05, ^∗∗^ = *p* < 0.01, ^∗∗∗^ = *p* < 0.001) between wild-type and mutant strains.

**FIGURE 5:**
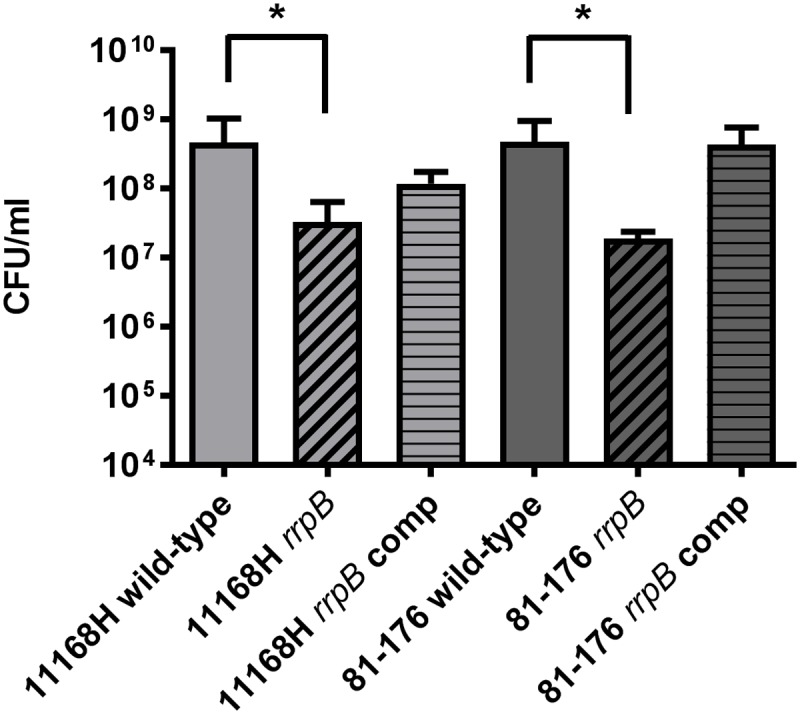
**Effect of oxidative stress on the survival of *C. jejuni* wild-type strains 11168H and 81-176, *rrpB* mutants and *rrpB* complements**. *C. jejuni* strains were incubated with 25 mM H_2_O_2_ for 15 min at 37°C under microaerobic conditions. Bacterial survival was subsequently assessed. Asterisks denote a statistically significant difference (^∗^ = *p* < 0.05) between wild-type and mutant strains.

To further investigate the link between oxidative stress resistance and the varied distribution of *rrpA* and *rrpB*, *rrpA* and *rrpB* single mutants were constructed in the 11168H and 81-176 wild-type strains (*rrpB*^+^) and *rrpA* mutants were also constructed in the 81116 and M1 wild-type strains (*rrpB*^-^). The mutants obtained did not have any observable deficiency in motility or growth rate under standard conditions (data not shown). Compared to the respective wild-type strains, both the 11168H and 81-176 *rrpA* mutants exhibited an increased sensitivity to 25 mM H_2_O_2_, however, the 81116 and M1 *rrpA* mutants exhibited wild-type levels of survival (**Figure 4**). Neither the 11168H and 81-176 wild-type strains nor the respective *rrpA* mutants survived exposure to higher concentrations of H_2_O_2_ (50 or 100 mM), however, the 81116 and M1 wild-type strains and the respective *rrpA* mutants all exhibited only slightly increased sensitivity to these higher concentrations of H_2_O_2_ (**Figure 4**). 11168H and 81-176 *rrpB* mutants exhibited the same increased sensitivity to 25 mM H_2_O_2_ as the *rrpA* mutants (**Figure 5**), but did not survive the higher concentrations of H_2_O_2_ (data not shown). Cumene hydroperoxide and menadione stress assays were also performed on the 11168H, 81-176, 81116, M1 wild-type strains and the respective *rrpA* and *rrpB* mutants (Supplementary Figure 3). There were no significant differences in sensitivity to either cumene hydroperoxide or menadione between any of the wild-type strains and respective mutants, with the exception that the 81116 *rrpA* mutant appeared to be more resistant to cumene hydroperoxide stress than the 81116 wild-type strain.

### Mutation of *rrpA* Reduces Growth under Aerobic Stress Conditions in *rrpB*^+^ Strains, But Not in *rrpB^-^* Strains

**FIGURE 6:**
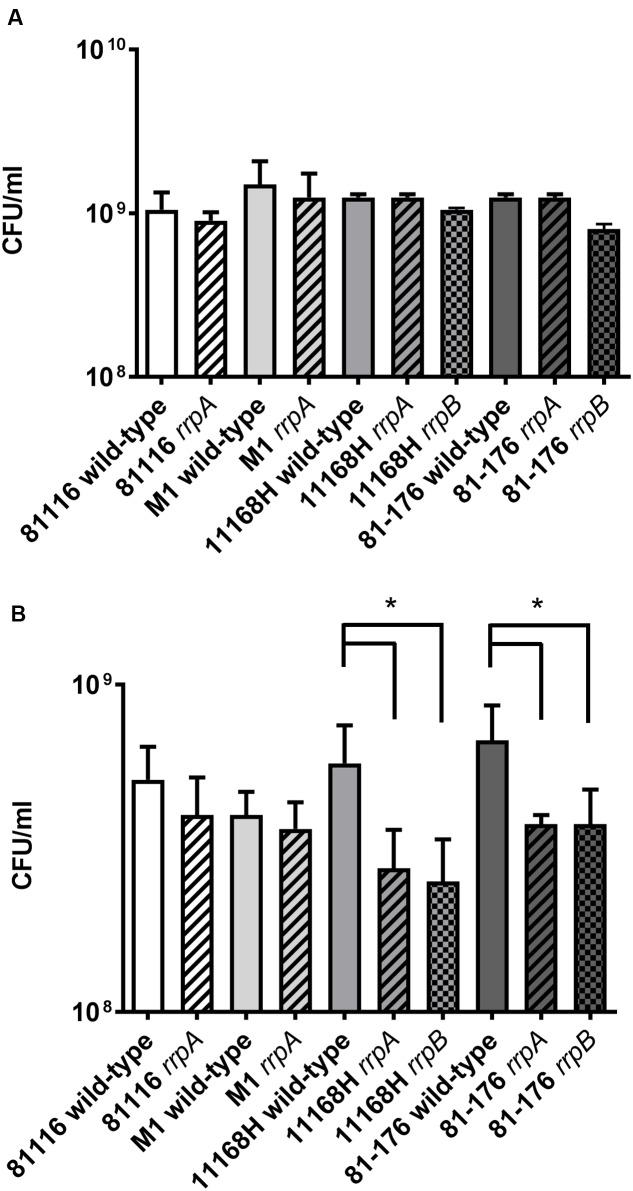
**Growth assays on *C. jejuni* 11168H, 81-176, 81116 and M1 wild-type strains and respective *rrpA and rrpB* mutants**. Brucella broth was pre-incubated at 37°C under microaerobic conditions for 24 h, then inoculated with a bacterial suspension to a final OD_600_ of 0.1 and grown for 16 h with shaking at 75 rpm at 37°C under either microaerobic conditions **(A)** or aerobic conditions **(B)**. Bacterial CFU were also assessed at 16 h. Asterisks denote a statistically significant difference (^∗^ = *p* < 0.05) between wild-type and mutant strains.

In *C. jejuni* 11168H, *rrpA* and *rrpB* mutants exhibit reduced growth under aerobic stress conditions (Gundogdu et al., 2011, 2015). The ability of *rrpA* and *rrpB* mutants to grow under aerobic stress conditions was compared to the respective four wild-type strains. No differences in bacterial growth kinetics (Supplementary Figure 4A) or in CFU counts after 16 h (**Figure 6A**) was observed under microaerobic conditions. However, differences were observed under aerobic conditions. All four *rrpA* mutants displayed significantly reduced growth kinetics during the logarithmic phase under aerobic conditions (Supplementary Figure 4B). However, compared to the respective wild-type strains, only the 11168H and 81-176 *rrpA* mutants exhibited a reduction in CFU counts after 16 h, whilst the 81116 and M1 *rrpA* mutants did not (**Figure 6B**).

### Mutation of *rrpA* Results in Decreased Cytotoxicity in the *Galleria mellonella* Larvae Model of Infection

**FIGURE 7:**
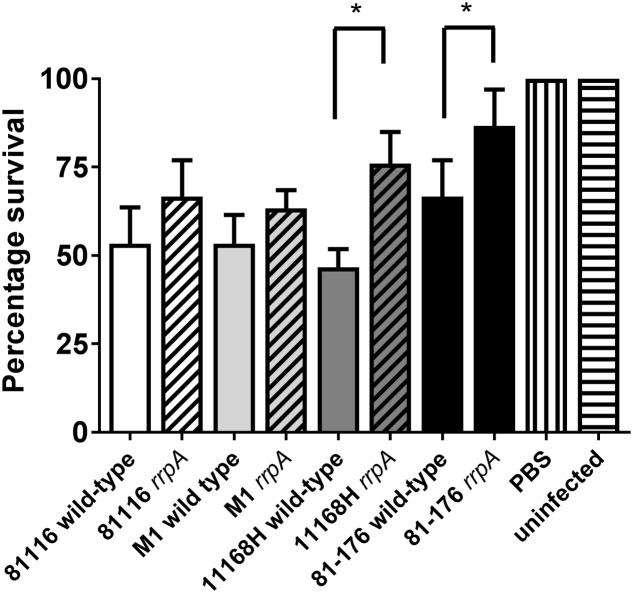
***Galleria mellonella* infection model on *C. jejuni* 11168H, 81-176, 81116, and M1 wild-type strains and respective *rrpA and rrpB* mutants**. *G. mellonella* larvae were injected with a 10 μl inoculum of a 24 h *C. jejuni* culture diluted to OD_600_ 0.1 by micro-injection in the right foremost leg, giving an infectious dose of approximately 10^6^ CFU. Larvae were incubated at 37°C with survival and appearance recorded at 24 h intervals. PBS and no injection controls were used. For each experiment, 10 *G. mellonella* larvae were infected and experiments were repeated in triplicate. Asterisks denote a statistically significant difference (^∗^ = *p* < 0.05) between wild-type and mutant strains.

*Galleria mellonella* larvae have been used as a model to study infection with many different enteric pathogens including *C. jejuni* (Champion et al., 2009). Insect larvae possess specialized phagocytic cells, termed haemocytes (Bergin et al., 2005; Mylonakis et al., 2007). Haemocytes mimic the functions of phagocytic cells in mammals and are able to degrade bacterial pathogens as well as generate bactericidal compounds such as superoxide via a respiratory burst (Lavine and Strand, 2002; Bergin et al., 2005). Both 11168H *rrpA* and *rrpB* mutants have been shown to exhibit reduced cytotoxicity in this model of infection compared to the wild-type strain (Gundogdu et al., 2011, 2015). The cytotoxicity of these mutants in *G. mellonella* larvae compared to the respective wild-type strains was investigated. Infection with either the 11168H or 81-176 *rrpA* mutants resulted in a statistically significant decrease in cytotoxicity to *G. mellonella* larvae compared to infection with the respective wild-type strains (**Figure 7**). Infection with the 81116 or M1 *rrpA* mutants also resulted in a decrease in cytotoxicity to *G. mellonella* larvae compared to infection with the respective wild-type strains, although this decrease was not statistically significant (**Figure 7**).

### Mutation of *rrpA* Results in Increased Biofilm Formation

**FIGURE 8:**
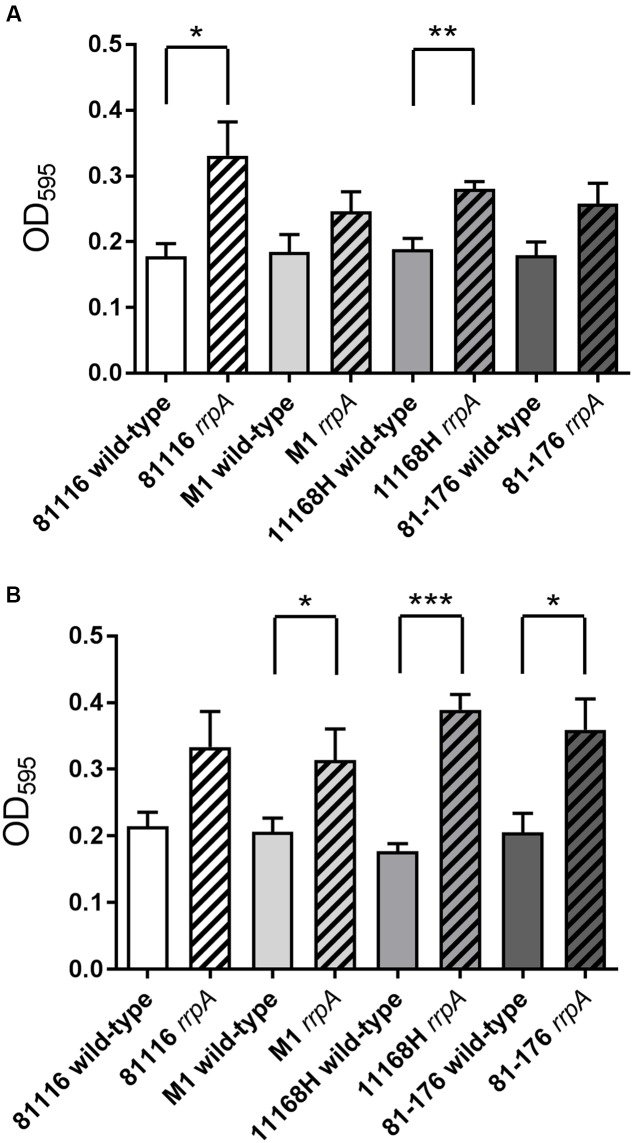
**Biofilm assay on *C. jejuni* 11168H, 81-176, 81116, and M1 wild-type strains and respective *rrpA and rrpB* mutants**. *C. jejuni* 11168H wild-type strain and mutants were grown for 72 h under aerobic **(A)** and microaerobic **(B)** growth conditions at 37°C without shaking, rinsed three times with PBS, followed by crystal violet staining. Asterisks denote a statistically significant difference (^∗^ = *p* < 0.05, ^∗∗^ = *p* < 0.01, ^∗∗∗^ = *p* < 0.001) between strains.

Several studies have demonstrated that *C. jejuni* exhibits increased biofilm formation under increased oxidative stress conditions (Fields and Thompson, 2008; Reuter et al., 2010). Previously *C. jejuni* 11168H *rrpA* and *rrpB* mutants exhibited an enhanced ability to form biofilms under both aerobic and microaerobic conditions (Gundogdu et al., 2015). All four *rrpA* mutants exhibited an increase in biofilm formation under both aerobic (**Figure 8A**) and microaerobic (**Figure 8B**) conditions after 72 h compared to the respective wild-type strains, which was statistically significant for the 11168H and 81116 *rrpA* mutants under aerobic conditions and for the 11168H, 81-176, and M1 *rrpA* mutants under microaerobic conditions. In addition both the 11168H *rrpB* mutant (Gundogdu et al., 2015) and 81-176 *rrpB* mutant (data not shown) exhibit increased biofilm formation under aerobic and microaerobic conditions when compared to the respective wild-type strains.

## Discussion

*Campylobacter jejuni* will be exposed to ROS both during colonization or infection of a host and in the environment as well as during the course of normal bacterial metabolism. It remains a conundrum as to how this microaerophilic pathogen is so widely dispersed, highly prevalent and able to survive in the ambient environment. *C. jejuni* contains a number of different mechanisms for counteracting the effects of oxidative stress and the control of the *C. jejuni* oxidative stress response is complex involving multiple inter-linked levels of regulation (Atack and Kelly, 2009). The re-annotation of the *C. jejuni* NCTC 11168 genome sequence (Gundogdu et al., 2007) identified both RrpA and RrpB as putative MarR-type transcriptional regulators which were subsequently shown to be involved in the *C. jejuni* peroxide and aerobic stress response (Gundogdu et al., 2011, 2015). All the other regulators of the *C. jejuni* oxidative stress response, such as PerR, Fur, CosR, CsrA, CprRS, and RacRS, are all conserved (>99.8%) amongst all the *C. jejuni* and *C. coli* wild-type strains in this study (van Vliet et al., 1999; Atack and Kelly, 2009; Palyada et al., 2009; Hwang et al., 2012). Analysis of the distribution of *rrpA* and *rrpB* in 3,746 *C. jejuni* and 486 *C. coli* genomes from public databases confirmed that the vast majority of *C. jejuni* strains contain *rrpA*, whilst the presence of *rrpB* is more restricted, with the distribution linked to MLST clonal complex. The majority of *C. jejuni* strains that contain both *rrpA* and *rrpB* are from ST-21 and ST-61. ST-21 strains are often associated with human infections (Sheppard et al., 2009) and ST-61 strains with infections of livestock (Kwan et al., 2008; Rotariu et al., 2009).

Analysis of the distribution of *rrpA* and *rrpB* from the microarray data study of 270 *C. jejuni* strains (Stabler et al., 2013) identified water and wildlife strains predominantly as *rrpB*^-^, whereas livestock-associated strains were predominantly *rrpB*^+^ (Supplementary Figure 1; Supplementary Table 1). This distribution of *rrpA* and *rrpB* was also reflected in the analysis of the whole genome sequence data (**Figure 1**) where red-labeled clonal complexes represent livestock-associated lineages and blue-labeled clonal complexes represent water and wildlife-associated lineages. It is, however, not always clear whether each clonal complex is associated with a single host as clonal complexes are regularly isolated from multiple animal species and thus attribution to a single host reservoir is difficult using MLST data alone (Dearlove et al., 2016). One example is the ST-48 complex that has been isolated from humans, cattle and sand from beaches (Dingle et al., 2002). Another example is ST-257 and ST-61 that have been isolated from chicken and ruminants, yet ST-257 is designated as a water and wildlife-associated lineage, whereas ST-61 is denoted as a livestock-associated lineage (Sheppard et al., 2011). These livestock and water and wildlife descriptions may be somewhat generalized and investigating the properties of individual STs will be more important to understand the source. ST-21, ST-206, and ST-48 may form a “complex group” of related genotypes that are widely distributed, perhaps reflecting the “generalist” ability to colonize a wide range of hosts (Dingle et al., 2002; Sheppard et al., 2011).

The location of *rrpA* and *rrpB* close to a type I Restriction-Modification (*hsd*) system suggests an explanation as to why *rrpA* is present in almost all *C. jejuni* strains, whilst the presence of *rrpB* is restricted and appears to correlate with MLST clonal complexes. Restriction-modification (R-M) systems are ubiquitous in the bacterial world and provide a defense against foreign DNA and bacteriophages (Wilson and Murray, 1991). Foreign DNA is cleaved by endonucleases but host DNA avoids damage due to methylation. R-M systems have been classified into four distinct groups, type I, type II, type III, and type IV. A type I R-M locus was identified in the genome sequence of NCTC 11168 (*Cj1549*–*Cj1553*) (Parkhill et al., 2000). The type I enzyme is a bi-functional, multi-subunit complex consisting of HsdR, HsdM, and HsdS. Further analysis of the type I R-M system from 73 *C. jejuni* strains (including NCTC 11168, 81-176, and 81116) assigned some *hsd* systems to the classical type IC family but also identified two additional type I R-M families, termed type IAB and type IF (Miller et al., 2005). 81116 contains a type IAB *hsd* system, whilst both NCTC 11168 and 81-176 contain a type IF *hsd* system. This study also found evidence for extensive rearrangements within the *C. jejuni hsd* loci of the same family but suggested that the low sequence similarity between the IC, IAB, and IF families would make inter-family recombination events unlikely. The NCTC 11168 version of the hypervariable central region identified in this study, which contains the type IF *hsd* system and *rrpB*, represents the majority of ST-21, ST-61, ST-42, and ST-353 strains. 81-176 also contains the type IF *hsd* system and *rrpB*. However, the 81116 version of this hypervariable central region, which represents the majority of all the other *C. jejuni* ST strains analyzed in this study, contains a type IAB *hsd* system and does not contain *rrpB*. The lack of recombination between *C. jejuni* strains containing the type IF *hsd* system and *rrpB* with other *C. jejuni* strains containing a different family of type I *hsd* system could be the explanation for the restricted distribution of *rrpB* amongst *C. jejuni* strains. This region of the *C. jejuni* genome has previously been identified as hypervariable region 14, one of 16 hypervariable regions (Taboada et al., 2004). The presence of intervening ORFs between *hsdR* and *hsdS* (referred to as *rlo* genes for R-linked ORF) and also between *hsdS* and *hsdM* (referred to as *mlo* genes for M-linked ORFs) are a distinct feature of the *C. jejuni hsd* loci (Miller et al., 2005). *H. pylori* ModH from the type III R-M system has been shown to have a regulatory role (Srikhanta et al., 2011) suggesting that R-M systems may also have a role in the regulation of virulence gene expression (Vasu and Nagaraja, 2013). Recently the same hypervariable region in *C. coli* has been shown to contain a novel streptomycin resistance gene (Olkkola et al., 2016). The genomics-based screening also showed that there are different alleles of RrpA and RrpB (Supplementary Figure 2) leading to a N-terminal truncation, a C-terminal truncation or both. The truncated version of RrpB was predominantly observed in isolates of the ST-353 clonal complex, while truncated versions of RrpA were rare in most clonal complexes, but dominant in ST-607 isolates (Supplementary Table 2). Future studies could utilize these truncated versions to test for the role of the N-terminal and C-terminal regions in functionality of RrpA and RrpB.

*Campylobacter jejuni rrpB*^-^ strains exhibit a pattern of greater resistance to peroxide stress when compared to *rrpB*^+^ strains. This pattern was the same when comparing the 11168H, 81-176, 81116, and M1 *rrpA* mutants against the respective wild-type strain where *rrpA* mutants in *rrpB*^-^ strains were more resistant to peroxide stress compared to *rrpA* mutants in *rrpB*^+^ strains. In addition, when comparing bacterial growth of 11168H, 81-176, 81116, and M1 *rrpA* mutants against the respective wild-type strain under aerobic conditions, all four *rrpA* mutants displayed a reduced growth rate during the logarithmic phase. Both the 11168H and 81-176 *rrpA* mutants exhibited lower CFUs at 16 h compared to the respective wild-type strains. Even though both 81116 and M1 *rrpA* mutants also displayed slower growth rates compared to the respective wild-type strains when comparing respective CFU counts at 16 h, these differences were not statistically significant. The general pattern that *rrpB*^+^ strains exhibit increased sensitivity to peroxide and aerobic stresses is rather counterintuitive. Possibly having only a single Rrp regulator is more efficient in responding to such stresses. Certainly *rrpA* is the most conserved amongst *C. jejuni* strains. Even though the oxidative stress assays were performed on a relatively small number of selected strains, it is interesting to speculate why livestock-associated strains such as NCTC 11168 (ST-21) would be more sensitive to peroxide and aerobic stress, whilst water and wildlife-associated strains such as 81116 (ST-283) and M1 (ST-45) would be more resistance to such stresses is difficult to explain. One possible hypothesis is that the ability to survive aerobic stress is more important for the latter strains. The variation in the presence of *rrpA* and *rrpB* between different wild-type strains may play an important role in the ability of *C. jejuni* to adapt and survive in different environmental niches.

Infection with *rrpA* mutants from both *rrpB*^+^ and *rrpB*^-^ strains resulted in an increase in survival of *G. mellonella* larvae compared to infection with the respective wild-type strains. We have previously demonstrated that infection of *G. mellonella* with the 11168H *rrpA* mutant leads to increased survival of the *G. mellonella* larvae (Gundogdu et al., 2015). Here we demonstrate that the 81-176 *rrpA* mutant (*rrpB*^+^) also exhibits a similar statistically significant reduction in larval cytotoxicity. Both 81116 and M1 *rrpA* mutants (*rrpB*^-^) exhibit reduced larval cytotoxicity, but not to a significant degree. This suggests the *rrpA* mutants (like the *rrpB* mutants) are more susceptible to the host immune mechanisms resulting in reduced bacterial survival within *G. mellonella*. This also suggests that RrpA may in fact play a role in the oxidative stress response of *rrpB*^-^ strains such as 81116 and M1, but at a different level compared to *rrpB*^+^ strains, such as 11168H and 81-176. Certainly the enhanced resistance of the 81116 and M1 *rrpA* mutants to peroxide stress compared to the 11168H and 81-176 *rrpA* mutants is not reflected by increased virulence in the *G. mellonella* larvae infection model.

*Campylobacter jejuni* forms biofilms (Joshua et al., 2006; Gundogdu et al., 2011) and this may be an important factor in the survival of *C. jejuni* both within hosts and in the environment. Though our understanding of the specific mechanisms underlying biofilm formation in *C. jejuni* is still limited (Svensson et al., 2008), *C. jejuni* lacks the classical two component regulatory systems involved in biofilm formation that are present in other bacteria such as GacSA in *Pseudomonas aeruginosa* (Parkins et al., 2001). Biofilm formation has been linked to responses to oxidative and aerobic stress as *C. jejuni* biofilm formation is increased under aerobic conditions (Reuter et al., 2010) and mutation of genes encoding oxidative stress response proteins results in changes in biofilm formation (Oh and Jeon, 2014; Gundogdu et al., 2015). Analysis of biofilm formation demonstrated that *rrpA* mutants from both *rrpB*^+^ and *rrpB*^-^ strains exhibited an increased biofilm phenotype compared to the respective wild-type strain under both microaerobic and aerobic conditions. Again the enhanced resistance of the 81116 and M1 *rrpA* mutants to peroxide stress compared to the 11168H and 81-176 *rrpA* mutants is not reflected by a decrease in biofilm formation.

The basis of *C. jejuni* survival is dependent upon the ability to sense and respond to the different environments encountered within hosts and in the environment. In this study we identified bioinformatically that over 99% of *C. jejuni* strains contain *rrpA*, whilst *rrpB* is restricted and appears to correlate with livestock-associated MLST clonal complexes. There exists a conserved genetic structure for *rrpA*, whilst *rrpB* seems to be part of a transferable hypervariable region linked with variation in the type I R-M (*hsd*) system, giving an explanation for the more restricted distribution of *rrpB* amongst *C. jejuni* strains. *rrpB^-^* strains possess an increased level of resistance to peroxide and aerobic stress compared to *rrpB*^+^ strains. So whilst all the other oxidative stress response regulators such as PerR, Fur, CosR, CsrA, CprRS, RacRS, and RrpA appear to be conserved in *C. jejuni*, variation in the presence of RrpB between different wild-type strains may play an important role for altered oxidative stress responses through the concerted actions of these multiple regulators in this microaerophilic pathogen. This highlights the potential of genetic variation in the natural population in the adaptation to different environmental niches.

## Author Contributions

OG, DdS, BW, and ND designed this study. OG, DdS, BM, and AE performed all the lab-based experimental work. OG, DdS, and AvV performed all the bioinformatic analysis. OG, DdS, BW, AvV, and ND wrote the manuscript.

## Conflict of Interest Statement

The authors declare that the research was conducted in the absence of any commercial or financial relationships that could be construed as a potential conflict of interest.

## References

[B1] Atack J. M. Kelly D. J. (2009). Oxidative stress in *Campylobacter jejuni*: responses, resistance and regulation. *Future Microbiol.* 4 677–690. 10.2217/fmb.09.44 19659424

[B2] Bergin D. Reeves E. P. Renwick J. Wientjes F. B. Kavanagh K. (2005). Superoxide production in *Galleria mellonella* hemocytes: identification of proteins homologous to the NADPH oxidase complex of human neutrophils. *Infect. Immun.* 73 4161–4170. 10.1128/IAI.73.7.4161-4170.2005 15972506 PMC1168619

[B3] Brown H. L. Reuter M. Hanman K. Betts R. P. Van Vliet A. H. (2015). Prevention of biofilm formation and removal of existing biofilms by extracellular DNases of *Campylobacter jejuni*. *PLoS ONE* 10:e0121680 10.1371/journal.pone.0121680 PMC4372405 25803828

[B4] Byrne C. M. Clyne M. Bourke B. (2007). *Campylobacter jejuni* adhere to and invade chicken intestinal epithelial cells in vitro. *Microbiology* 153 561–569. 10.1099/mic.0.2006/000711-0 17259628

[B5] Champion O. L. Cooper I. A. James S. L. Ford D. Karlyshev A. Wren B. W. (2009). *Galleria mellonella* as an alternative infection model for *Yersinia pseudotuberculosis*. *Microbiology* 155 1516–1522. 10.1099/mic.0.026823-0 19383703

[B6] Champion O. L. Gaunt M. W. Gundogdu O. Elmi A. Witney A. A. Hinds J. (2005). Comparative phylogenomics of the food-borne pathogen *Campylobacter jejuni* reveals genetic markers predictive of infection source. *Proc. Natl. Acad. Sci. U.S.A* 102 16043–16048. 10.1073/pnas.0503252102 16230626 PMC1276044

[B7] Champion O. L. Karlyshev A. V. Senior N. J. Woodward M. La Ragione R. Howard S. L. (2010). Insect infection model for *Campylobacter jejuni* reveals that O-methyl phosphoramidate has insecticidal activity. *J. Infect. Dis.* 201 776–782. 10.1086/650494 20113177

[B8] Cody A. J. Mccarthy N. D. Jansen Van Rensburg M. Isinkaye T. Bentley S. D. Parkhill J. (2013). Real-time genomic epidemiological evaluation of human *Campylobacter* isolates by use of whole-genome multilocus sequence typing. *J. Clin. Microbiol.* 51 2526–2534. 10.1128/JCM.00066-13 23698529 PMC3719633

[B9] D’Autreaux B. Toledano M. B. (2007). ROS as signalling molecules: mechanisms that generate specificity in ROS homeostasis. *Nat. Rev. Mol. Cell Biol.* 8 813–824. 10.1038/nrm2256 17848967

[B10] Dearlove B. L. Cody A. J. Pascoe B. Meric G. Wilson D. J. Sheppard S. K. (2016). Rapid host switching in generalist *Campylobacter* strains erodes the signal for tracing human infections. *ISME J.* 10 721–729. 10.1038/ismej.2015.149 26305157 PMC4677457

[B11] Dingle K. E. Colles F. M. Ure R. Wagenaar J. A. Duim B. Bolton F. J. (2002). Molecular characterization of *Campylobacter jejuni* clones: a basis for epidemiologic investigation. *Emerg. Infect. Dis.* 8 949–955. 10.3201/eid0809.020122 12194772 PMC2732546

[B12] Dwivedi R. Nothaft H. Garber J. Xin Kin L. Stahl M. Flint A. (2016). L-fucose influences chemotaxis and biofilm formation in *Campylobacter jejuni*. *Mol. Microbiol.* 101 575–589. 10.1111/mmi.13409 27145048

[B13] Fang F. C. (2004). Antimicrobial reactive oxygen and nitrogen species: concepts and controversies. *Nat. Rev. Microbiol.* 2 820–832. 10.1038/nrmicro1004 15378046

[B14] Fields J. A. Thompson S. A. (2008). *Campylobacter jejuni* CsrA mediates oxidative stress responses, biofilm formation, and host cell invasion. *J. Bacteriol.* 190 3411–3416. 10.1128/JB.01928-07 18310331 PMC2347403

[B15] Friis C. Wassenaar T. M. Javed M. A. Snipen L. Lagesen K. Hallin P. F. (2010). Genomic characterization of *Campylobacter jejuni* strain M1. *PLoS ONE* 5:e12253 10.1371/journal.pone.0012253 PMC2928727 20865039

[B16] Gundogdu O. Bentley S. D. Holden M. T. Parkhill J. Dorrell N. Wren B. W. (2007). Re-annotation and re-analysis of the *Campylobacter jejuni* NCTC11168 genome sequence. *BMC Genomics* 8:162 10.1186/1471-2164-8-162 PMC1899501 17565669

[B17] Gundogdu O. Da Silva D. T. Mohammad B. Elmi A. Mills D. C. Wren B. W. (2015). The *Campylobacter jejuni* MarR-like transcriptional regulators RrpA and RrpB both influence bacterial responses to oxidative and aerobic stresses. *Front. Microbiol.* 6:724 10.3389/fmicb.2015.00724 PMC4508579 26257713

[B18] Gundogdu O. Mills D. C. Elmi A. Martin M. J. Wren B. W. Dorrell N. (2011). The *Campylobacter jejuni* transcriptional regulator Cj1556 plays a role in the oxidative and aerobic stress response and is important for bacterial survival in vivo. *J. Bacteriol.* 193 4238–4249. 10.1128/JB.05189-11 21642451 PMC3147681

[B19] Handley R. A. Mulholland F. Reuter M. Ramachandran V. K. Musk H. Clissold L. (2015). PerR controls oxidative stress defence and aerotolerance but not motility-associated phenotypes of *Campylobacter jejuni*. *Microbiology* 161 1524–1536. 10.1099/mic.0.000109 25968890

[B20] Hwang S. Kim M. Ryu S. Jeon B. (2011). Regulation of oxidative stress response by CosR, an essential response regulator in *Campylobacter jejuni*. *PLoS ONE* 6:e22300 10.1371/journal.pone.0022300 PMC3139631 21811584

[B21] Hwang S. Zhang Q. Ryu S. Jeon B. (2012). Transcriptional regulation of the CmeABC multidrug eﬄux pump and the KatA catalase by CosR in *Campylobacter jejuni*. *J. Bacteriol.* 194 6883–6891. 10.1128/JB.01636-12 23065977 PMC3510603

[B22] Imlay J. A. (2003). Pathways of oxidative damage. *Annu. Rev. Microbiol.* 57 395–418. 10.1146/annurev.micro.57.030502.090938 14527285

[B23] Jolley K. A. Maiden M. C. (2010). BIGSdb: scalable analysis of bacterial genome variation at the population level. *BMC Bioinformatics* 11:595 10.1186/1471-2105-11-595 PMC3004885 21143983

[B24] Jones M. A. Marston K. L. Woodall C. A. Maskell D. J. Linton D. Karlyshev A. V. (2004). Adaptation of *Campylobacter jejuni* NCTC11168 to high-level colonization of the avian gastrointestinal tract. *Infect. Immun.* 72 3769–3776. 10.1128/IAI.72.7.3769-3776.2004 15213117 PMC427441

[B25] Joshua G. W. Guthrie-Irons C. Karlyshev A. V. Wren B. W. (2006). Biofilm formation in *Campylobacter jejuni*. *Microbiology* 152 387–396. 10.1099/mic.0.28358-0 16436427

[B26] Karlyshev A. V. Linton D. Gregson N. A. Wren B. W. (2002). A novel paralogous gene family involved in phase-variable flagella-mediated motility in *Campylobacter jejuni*. *Microbiology* 148 473–480. 10.1099/00221287-148-2-473 11832511

[B27] Kim J. C. Oh E. Kim J. Jeon B. (2015). Regulation of oxidative stress resistance in *Campylobacter jejuni*, a microaerophilic foodborne pathogen. *Front. Microbiol.* 6:751 10.3389/fmicb.2015.00751 PMC4518328 26284041

[B28] Korlath J. A. Osterholm M. T. Judy L. A. Forfang J. C. Robinson R. A. (1985). A point-source outbreak of *campylobacter*iosis associated with consumption of raw milk. *J. Infect. Dis.* 152 592–596. 10.1093/infdis/152.3.592 4031557

[B29] Kruczkiewicz P. Mutschall S. Barker D. Thomas J. E. Domselaar G. V. H. Gannon V. (2013). “MIST: a tool for rapid in silico generation of molecular data from bacterial genome sequences,” in *Proceedings of the 4th International Conference on Bioinformatics Models, Methods and Algorithms*, Barcelona, 316–323.

[B30] Kwan P. S. Birtles A. Bolton F. J. French N. P. Robinson S. E. Newbold L. S. (2008). Longitudinal study of the molecular epidemiology of *Campylobacter jejuni* in cattle on dairy farms. *Appl. Environ. Microbiol.* 74 3626–3633. 10.1128/AEM.01669-07 18424539 PMC2446552

[B31] Lavine M. D. Strand M. R. (2002). Insect hemocytes and their role in immunity. *Insect Biochem. Mol. Biol.* 32 1295–1309. 10.1016/S0965-1748(02)00092-9 12225920

[B32] Miller W. G. Pearson B. M. Wells J. M. Parker C. T. Kapitonov V. V. Mandrell R. E. (2005). Diversity within the *Campylobacter jejuni* type I restriction-modification loci. *Microbiology* 151 337–351. 10.1099/mic.0.27327-0 15699185

[B33] Mylonakis E. Casadevall A. Ausubel F. M. (2007). Exploiting amoeboid and non-vertebrate animal model systems to study the virulence of human pathogenic fungi. *PLoS Pathog.* 3:e101 10.1371/journal.ppat.0030101 PMC1933451 17676994

[B34] Oh E. Jeon B. (2014). Role of alkyl hydroperoxide reductase (AhpC) in the biofilm formation of *Campylobacter jejuni*. *PLoS ONE* 9:e87312 10.1371/journal.pone.0087312 PMC3909096 24498070

[B35] Olkkola S. Culebro A. Juntunen P. Hanninen M. L. Rossi M. (2016). Functional genomics in *Campylobacter coli* identified a novel streptomycin resistance gene located in a hypervariable genomic region. *Microbiology* 162 1157–1166. 10.1099/mic.0.000304 27154456

[B36] Page A. J. Cummins C. A. Hunt M. Wong V. K. Reuter S. Holden M. T. (2015). Roary: rapid large-scale prokaryote pan genome analysis. *Bioinformatics* 31 3691–3693. 10.1093/bioinformatics/btv421 26198102 PMC4817141

[B37] Palyada K. Sun Y. Q. Flint A. Butcher J. Naikare H. Stintzi A. (2009). Characterization of the oxidative stress stimulon and PerR regulon of *Campylobacter jejuni*. *BMC Genomics* 10:481 10.1186/1471-2164-10-481 PMC2772861 19835633

[B38] Parkhill J. Wren B. W. Mungall K. Ketley J. M. Churcher C. Basham D. (2000). The genome sequence of the food-borne pathogen *Campylobacter jejuni* reveals hypervariable sequences. *Nature* 403 665–668. 10.1038/35001088 10688204

[B39] Parkins M. D. Ceri H. Storey D. G. (2001). *Pseudomonas aeruginosa* GacA, a factor in multihost virulence, is also essential for biofilm formation. *Mol. Microbiol.* 40 1215–1226. 10.1046/j.1365-2958.2001.02469.x 11401724

[B40] Pearson B. M. Louwen R. Van Baarlen P. Van Vliet A. H. (2015). Differential Distribution of Type II CRISPR-Cas systems in agricultural and nonagricultural *Campylobacter coli* and *Campylobacter jejuni* isolates correlates with lack of shared environments. *Genome Biol. Evol.* 7 2663–2679. 10.1093/gbe/evv174 26338188 PMC4607530

[B41] Reuter M. Mallett A. Pearson B. M. Van Vliet A. H. (2010). Biofilm formation by *Campylobacter jejuni* is increased under aerobic conditions. *Appl. Environ. Microbiol.* 76 2122–2128. 10.1128/AEM.01878-09 20139307 PMC2849235

[B42] Rotariu O. Dallas J. F. Ogden I. D. Macrae M. Sheppard S. K. Maiden M. C. (2009). Spatiotemporal homogeneity of *Campylobacter* subtypes from cattle and sheep across northeastern and southwestern Scotland. *Appl. Environ. Microbiol.* 75 6275–6281. 10.1128/AEM.00499-09 19700557 PMC2753054

[B43] Ruiz-Palacios G. M. (2007). The health burden of *Campylobacter* infection and the impact of antimicrobial resistance: playing chicken. *Clin. Infect. Dis* 44 701–703. 10.1086/509936 17278063

[B44] Seemann T. (2014). Prokka: rapid prokaryotic genome annotation. *Bioinformatics* 30 2068–2069. 10.1093/bioinformatics/btu153 24642063

[B45] Sheppard S. K. Colles F. M. Mccarthy N. D. Strachan N. J. Ogden I. D. Forbes K. J. (2011). Niche segregation and genetic structure of *Campylobacter jejuni* populations from wild and agricultural host species. *Mol. Ecol.* 20 3484–3490. 10.1111/j.1365-294X.2011.05179.x 21762392 PMC3985062

[B46] Sheppard S. K. Dallas J. F. Strachan N. J. Macrae M. Mccarthy N. D. Wilson D. J. (2009). *Campylobacter* genotyping to determine the source of human infection. *Clin. Infect. Dis* 48 1072–1078. 10.1086/597402 19275496 PMC3988352

[B47] Srikhanta Y. N. Gorrell R. J. Steen J. A. Gawthorne J. A. Kwok T. Grimmond S. M. (2011). Phasevarion mediated epigenetic gene regulation in *Helicobacter pylori*. *PLoS ONE* 6:e27569 10.1371/journal.pone.0027569 PMC3230613 22162751

[B48] Stabler R. A. Larsson J. T. Al-Jaberi S. Nielsen E. M. Kay E. Tam C. C. (2013). Characterization of water and wildlife strains as a subgroup of *Campylobacter jejuni* using DNA microarrays. *Environ. Microbiol.* 15 2371–2383. 10.1111/1462-2920.12111 23530835

[B49] Svensson S. L. Frirdich E. Gaynor E. C. (2008). “Survival strategies of *Campylobacter jejuni*: stress responses, the viable but nonculturable state, and biofilms,” in *Campylobacter*, 3rd Edn, eds Nachmkin I. Szymanski C. M. Blaser M. J. (Washington, DC: ASM Press), 571–590.

[B50] Taboada E. N. Acedillo R. R. Carrillo C. D. Findlay W. A. Medeiros D. T. Mykytczuk O. L. (2004). Large-scale comparative genomics meta-analysis of *Campylobacter jejuni* isolates reveals low level of genome plasticity. *J. Clin. Microbiol.* 42 4566–4576. 10.1128/jcm.42.10.4566-4576.2004 15472310 PMC522315

[B51] Trieu-Cuot P. Gerbaud G. Lambert T. Courvalin P. (1985). In vivo transfer of genetic information between gram-positive and gram-negative bacteria. *EMBO J.* 4 3583–3587. 3937729 10.1002/j.1460-2075.1985.tb04120.x PMC554700

[B52] van Vliet A. H. Baillon M. L. Penn C. W. Ketley J. M. (1999). *Campylobacter jejuni* contains two fur homologs: characterization of iron-responsive regulation of peroxide stress defense genes by the PerR repressor. *J. Bacteriol.* 181 6371–6376. 10515927 10.1128/jb.181.20.6371-6376.1999 PMC103772

[B53] Van Vliet A. H. Kusters J. G. (2015). Use of alignment-free phylogenetics for rapid genome sequence-based typing of *Helicobacter pylori* virulence markers and antibiotic susceptibility. *J. Clin. Microbiol.* 53 2877–2888. 10.1128/JCM.01357-15 26135867 PMC4540916

[B54] van Vliet A. H. Rock J. D. Madeleine L. N. Ketley J. M. (2000). The iron-responsive regulator Fur of *Campylobacter jejuni* is expressed from two separate promoters. *FEMS Microbiol. Lett.* 188 115–118. 10.1111/j.1574-6968.2000.tb09180.x 10913692

[B55] van Vliet A. H. Wooldridge K. G. Ketley J. M. (1998). Iron-responsive gene regulation in a *Campylobacter jejuni* fur mutant. *J. Bacteriol.* 180 5291–5298. 9765558 10.1128/jb.180.20.5291-5298.1998 PMC107575

[B56] Vasu K. Nagaraja V. (2013). Diverse functions of restriction-modification systems in addition to cellular defense. *Microbiol. Mol. Biol. Rev.* 77 53–72. 10.1128/MMBR.00044-12 23471617 PMC3591985

[B57] Wassenaar T. M. Bleumink-Pluym N. M. Van Der Zeijst B. A. (1991). Inactivation of *Campylobacter jejuni* flagellin genes by homologous recombination demonstrates that flaA but not flaB is required for invasion. *EMBO J.* 10 2055–2061. 2065653 10.1002/j.1460-2075.1991.tb07736.x PMC452888

[B58] Wilson G. G. Murray N. E. (1991). Restriction and modification systems. *Annu. Rev. Genet.* 25 585–627. 10.1146/annurev.ge.25.120191.003101 1812816

[B59] Young K. T. Davis L. M. Dirita V. J. (2007). *Campylobacter jejuni*: molecular biology and pathogenesis. *Nat. Rev. Microbiol.* 5 665–679. 10.1038/nrmicro1718 17703225

