# Supplementary material for: The Campylobacter jejuni Oxidative Stress Regulator RrpB Is Associated with a Genomic Hypervariable Region and Altered Oxidative Stress Resistance
Source: Front Microbiol. 2016 Dec 26;7:2117. doi: 10.3389/fmicb.2016.02117 (PMC5183652; doi:10.3389/fmicb.2016.02117)
Supplement: Supplementary file 1 [file Table_1.DOCX]

**Supplementary Table 1. Presence of *rrpA* and *rrpB* in livestock and water & wildlife subclades**

| **Livestock** | **Total Strains** | ***rrpA* Only** | ***rrpB* Only** | ***rrpA* & *rrpB*** | **Neither** |
| --- | --- | --- | --- | --- | --- |
| **C6** | **23** | 20 (86.96%) |  |  | 3 (13.04%) |
| **C5** | **19** | 5 (26.32%) |  | 14 (73.68%) |  |
| **C4** | **13** | 1 (7.69%) |  | 12 (92.31% |  |
| **C3** | **21** |  |  | 21 (100.00%) |  |
| **C2** | **27** |  |  | 27 (100.00%) |  |
| **C1** | **30** | 2 (6.67%) | 1 (3.33%) | 27 (90.00%) |  |
| **Total** | **133** | **28 (21.05%)** | **1 (0.75%)** | **101 (75.94%)** | **3 (2.26%)** |
| **Water and Wildlife** |  |  |  |  |  |
| **C7** | **25** | 24 (96.00%) |  | 1 (4.00%) |  |
| **C8** | **39** | 35 (89.74%) |  | 4 (10.26%) |  |
| **C9i** | **15** | 7 (46.67%) |  | 5 (33.33%) | 3 (20.00%) |
| **C9ii** | **58** | 45 (77.59%) |  | 9 (15.52%) | 4 (6.90%) |
| **Total** | **137** | **111 (81.02%)** |  | **19 (13.87%)** | **7 (5.11%)** |
