# Supplementary material for: The Campylobacter jejuni Oxidative Stress Regulator RrpB Is Associated with a Genomic Hypervariable Region and Altered Oxidative Stress Resistance
Source: Front Microbiol. 2016 Dec 26;7:2117. doi: 10.3389/fmicb.2016.02117 (PMC5183652; doi:10.3389/fmicb.2016.02117)
Supplement: Supplementary file 4 [file Image_1.PDF]

Supplementary Figure 1

Presence of *rrpA* and *rrpB* amongst different *C. jejuni* sub-clades (C1-C9). Comparative pylogenomics based on microarray data of 270 *C. jejuni* isolates presented as a rooted phylogram to display clusters. Human clinical isolates are indicated by black branches, wildlife and water associated isolates by green branches, mammalian livestock isolates by blue branches, avian livestock isolates by red branches and dashed black branch indicates an indeterminate source. The percentage of isolates within each sub-clade containing only *rrpA* or both *rrpA* and *rrpB* is indicated. Figure adapted from Stabler *et al.* (2013).

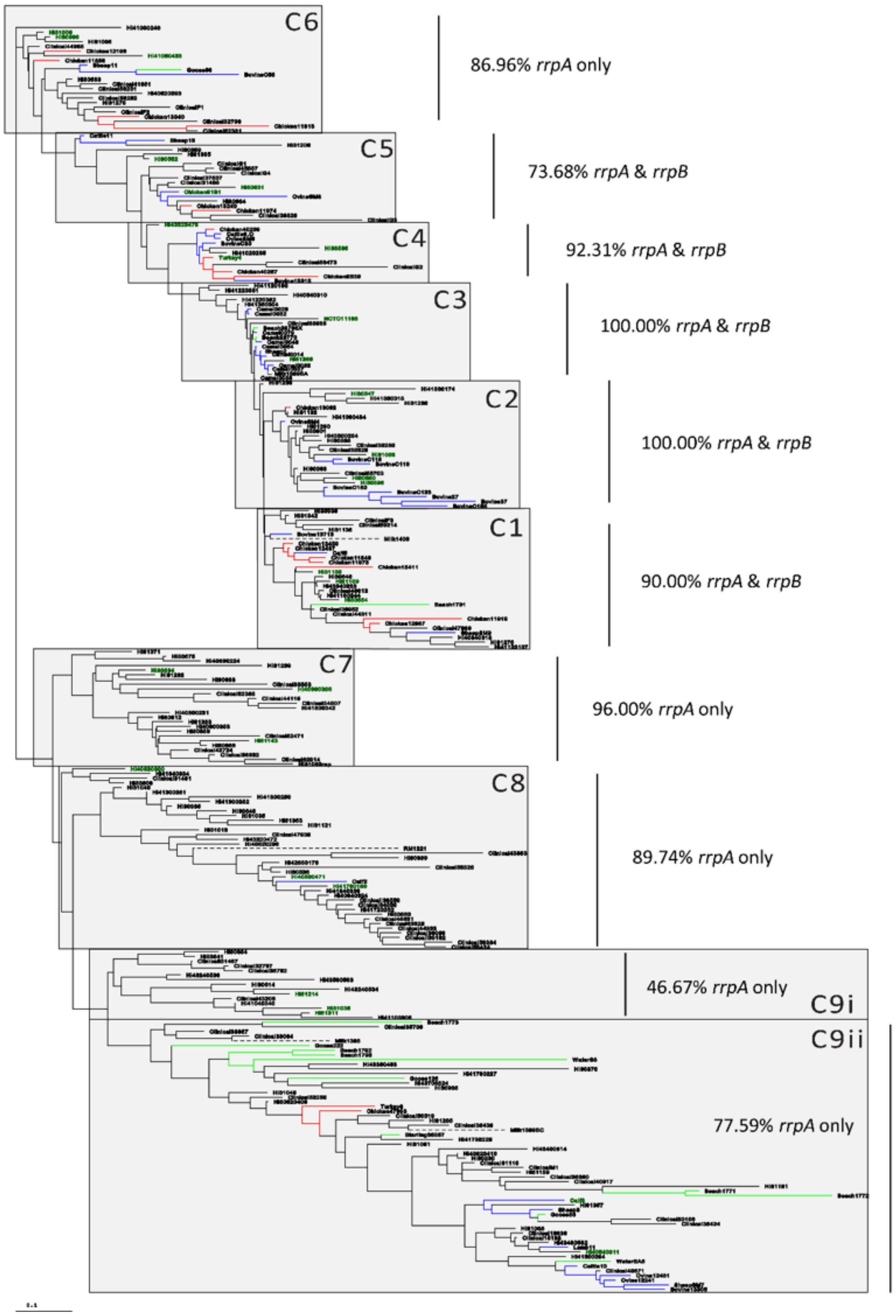
C6
86.96% *rrpA* only
C5
73.68% *rrpA* & *rrpB*
C4
92.31% *rrpA* & *rrpB*
C3
100.00% *rrpA* & *rrpB*
C2
100.00% *rrpA* & *rrpB*
C1
90.00% *rrpA* & *rrpB*
C7
96.00% *rrpA* only
C8
89.74% *rrpA* only
46.67% *rrpA* only
C9i
C9ii
77.59% *rrpA* only
