# Supplementary material for: The Campylobacter jejuni Oxidative Stress Regulator RrpB Is Associated with a Genomic Hypervariable Region and Altered Oxidative Stress Resistance
Source: Front Microbiol. 2016 Dec 26;7:2117. doi: 10.3389/fmicb.2016.02117 (PMC5183652; doi:10.3389/fmicb.2016.02117)
Supplement: Supplementary file 5 [file Image_2.PDF]

Supplementary Figure 2

RrpA full-length and truncated versions

```
CjNCTC11168_RrpA      -MTKENSPCNFEECGFNYTLALINGKYKMSILYCLFRYEIVRYNELKRFLSSISFKTLTN
Cj81116_RrpA          -MTKENSQCNFEECGFNYTLALINGKYKMSILYCLFRYEIVRYNELKRFLSSISFKTLTN
Cc76339_RrpA          VKNTKNSTCNYQECGFNYTLALISGKYKMSVLYCLYKDEIVRYNELNRILSPISFKTLTN
Cj414_RrpA            MKNTKNSTCNYQEYGFNYTLALISGKYKMSVLYCLYKDKIVRYNELKRILNPISFKTLTN
N-term_truncated      ----------------------------MSILYCLFRYEIVRYNELKRFLSSISFKTLTN
C-term_truncated      -MTKENSQCNFEECGFNYTLALINGKYKMSILYCLFRYEIVRYNELKRFLSSISFKTLTN
                                                  **:****:: :*******:*:*..********

CjNCTC11168_RrpA      TLRELENDGLIIRKEYAQIPPKVEYSLSKRGQSLIPILQAMSKWGKKDKKGKKCLN-
Cj81116_RrpA          TLRELENDGLIIRKEYAQIPPKVEYSLSKRGQSLIPILQAMCKWGEKDKKEKNA---
Cc76339_RrpA          VLRELESDGLITRKEYPQIPPKVEYSLSQKGQSFIPILQAMCDWGE-KNKRRIP---
Cj414_RrpA            VLRELENAGLIIRKEYPQIPPKVEYSLSKKGQSFIPILEAMCDWGRRKQKINILKIY
N-term_truncated      TLRELENDGLIIRKEYAQIPPKVEYSLSKRGQSLIPILQAMCDWEEENKKLQGK---
C-term_truncated      TLRELENDGLIIRKEYA----------------------------------------
                      .*****. *** ****.
```

RrpB full-length and truncated versions

```
CjNCTC11168_RrpB      MKKYHSLCPIETTLNLIGNKWKILIIRDLLQGTKRFGELRKSISFTKNQNISQNVLTQNL
Cc2544_RrpB           MKKYHSPCPVETTLNLIGNKWKILIIRELLDGEKRFGELRKNISATKNQNISQNVLTQNL
N-term_truncated      -----------------------------MQGTKRFGELRKSISFTKNQNISQNVLTQNL
N+C-term_truncated    -----------------------------MQGTKRFGELRKSISFTKNQNISQNVLTQNL
C-term_truncated      MKKYHSLCPIETTLNLIGNKWKILIIRDLLQGTKRFGELRKSISFTKNQNISQNVLTQNL
                                                   ::* ********.** ***************

CjNCTC11168_RrpB      RELEEAKLIKRKVYAEVPPKVEYSLTSLGNSLESILKSLENWGNSYKNIV
Cc2544_RrpB           RELEEAKLLKRKVYAEVPPRVEYSLTLLGSSLESVLKSLEIWGDKYKNMN
N-term_truncated      RELEEAKLIKRKVYAEVPPKVEYSLTSLGNSLESILKSLENWGNSYKNIV
N+C-term_truncated    RELEEAKLIKRKVYAEVPPKVEYLLISLGNSLESILKSLEN---------
C-term_truncated      REIRRSKTDQTQSLCRSSSKG-----------------------------
                      **:...:*  : :  .. ..:
```

Supplementary Figure 2. Presence of N- and C-terminal truncations in *C. jejuni* and *C. coli* RrpA and RrpB proteins. Amino acid sequences were obtained from *C. jejuni* NCTC 11168, 81116 and 414, and *C. coli* 2544 and 76339. Other truncated versions were obtained from genome sequences listed in Supplementary Table 2. Asterisks mean identical amino acids, colons and full stops show conservative substitutions. Alignments were made using ClustalX2. Yellow blocks indicate identical regions in all proteins, including regions with truncated proteins.
