# Supplementary material for: The Campylobacter jejuni Oxidative Stress Regulator RrpB Is Associated with a Genomic Hypervariable Region and Altered Oxidative Stress Resistance
Source: Front Microbiol. 2016 Dec 26;7:2117. doi: 10.3389/fmicb.2016.02117 (PMC5183652; doi:10.3389/fmicb.2016.02117)

Supplementary Figure 3A. Effect of cumene hydroperoxide oxidative stress on the survival of *C. jejuni* 11168H, 81-176, 81116 and M1 wild-type strains and respective *rrpA and rrpB* mutants. *C. jejuni* strains were incubated with 0.05% cumene hydroperoxide for 15 minutes at 37°C under microaerobic conditions. Bacterial survival was subsequently assessed. Asterisks denote a statistically significant difference (** = p <0.01) between control and tested strains.

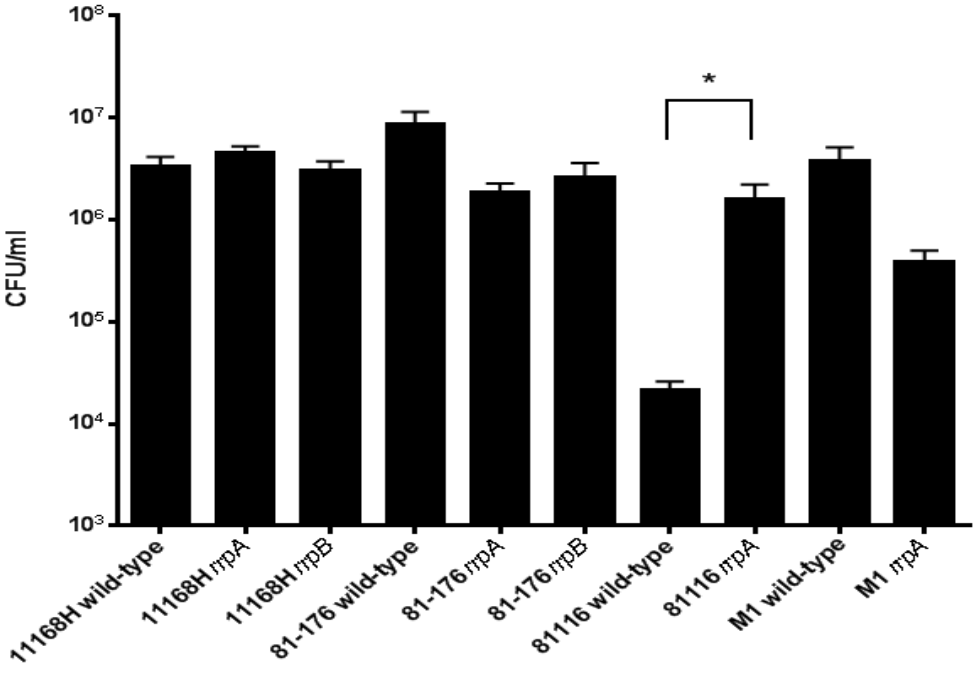

Supplementary Figure 3B. Effect of menadione oxidative stress on the survival of *C. jejuni* wild-type strains 11168H, 81-176, 81116, M1 and the respective *rrpA and rrpB* mutants. *C. jejuni* strains were incubated with 100 mM menadione for 1 h at 37°C under microaerobic conditions. Bacterial survival was subsequently assessed.

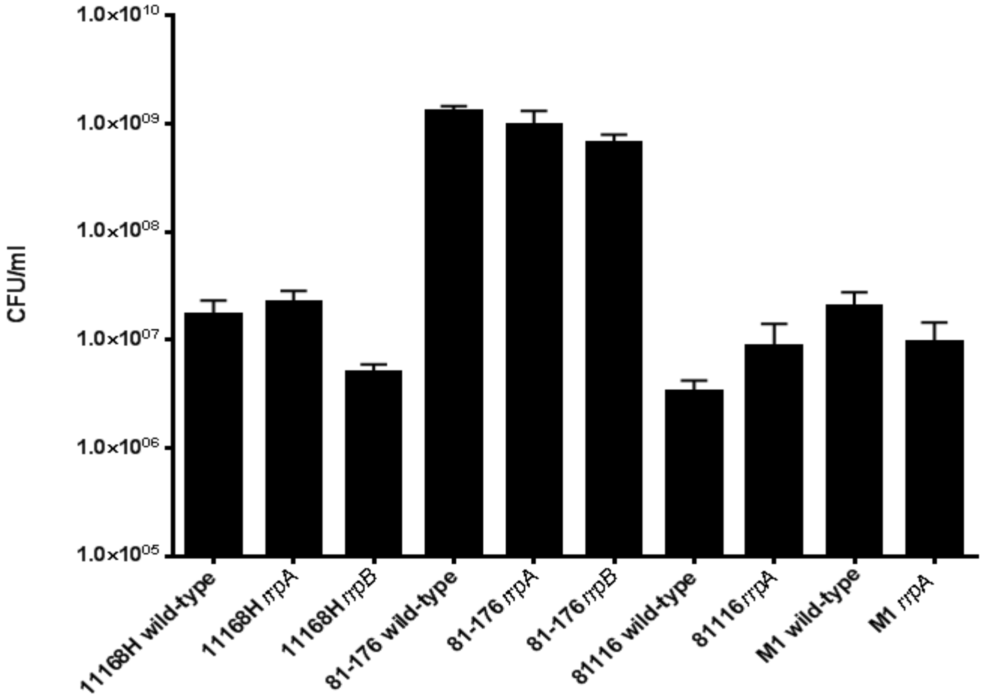

Supplement: Supplementary file 6 [file Image_3.PDF]
