# Supplementary material for: The Campylobacter jejuni Oxidative Stress Regulator RrpB Is Associated with a Genomic Hypervariable Region and Altered Oxidative Stress Resistance
Source: Front Microbiol. 2016 Dec 26;7:2117. doi: 10.3389/fmicb.2016.02117 (PMC5183652; doi:10.3389/fmicb.2016.02117)
Supplement: Supplementary file 7 [file Image_4.PDF]

Supplementary Figure 4. Growth curves for *C. jejuni* 11168H, 81-176, 81116 and M1 wild-type strain and the respective *rrpA* and *rrpB* mutant. Bacteria were grown under either microaerobic (A) or aerobic conditions (B) at 37°C (with shaking at 75 rpm) in Brucella broth with bacterial growth assessed by recording the OD_{600} of the culture at different time points. Asterisks denote a statistically significant difference (* = p <0.05).

A)

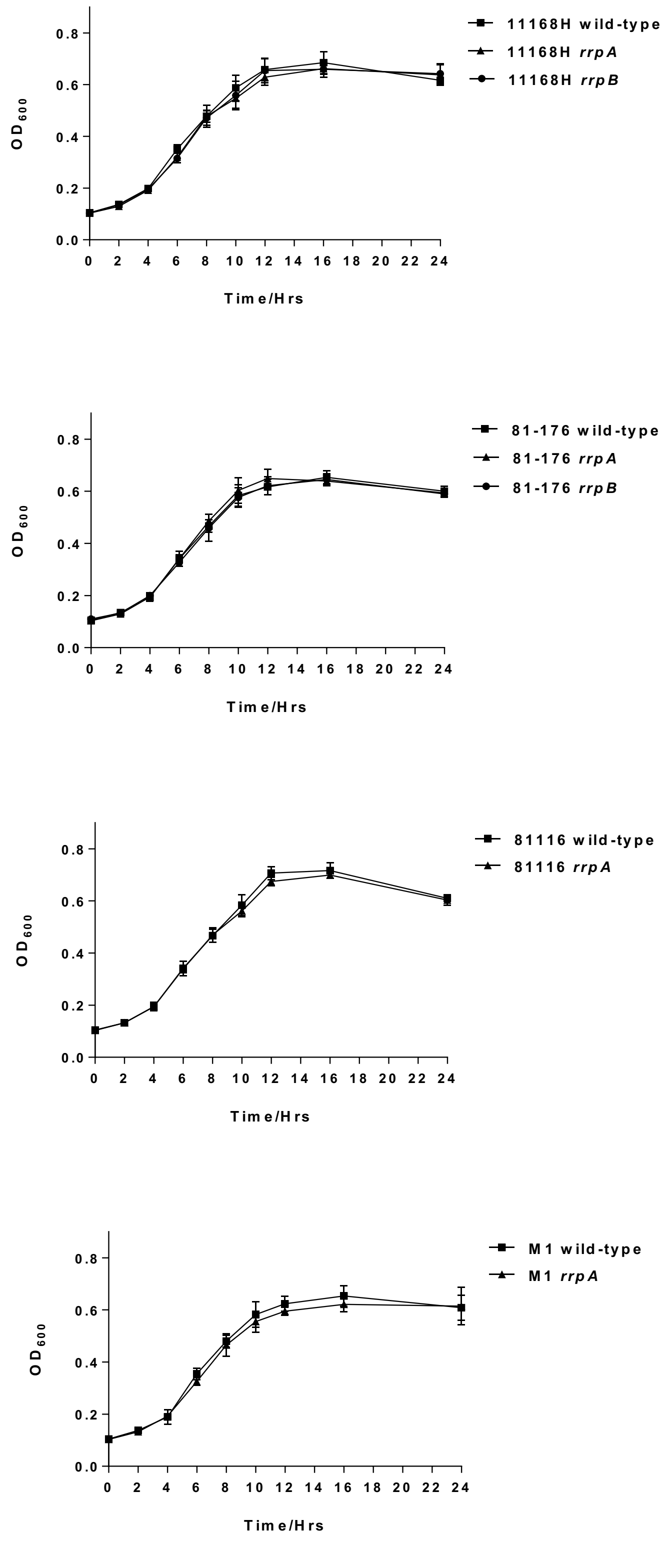

B)

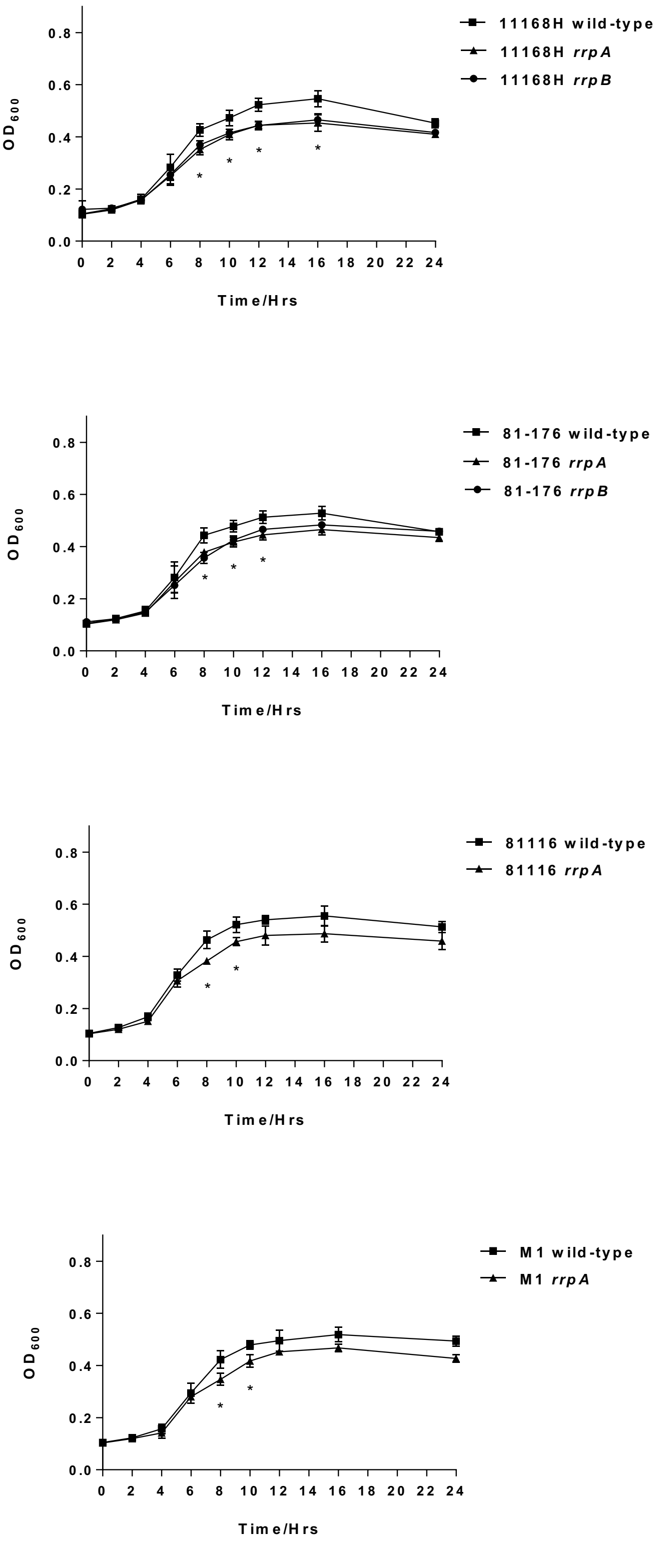
11168H wild-type
11168H rrpA
11168H rrpB
OD600
Time/Hrs
81-176 wild-type
81-176 rrpA
81-176 rrpB
OD600
Time/Hrs
81116 wild-type
81116 rrpA
OD600
Time/Hrs
M1 wild-type
M1 rrpA
OD600
Time/Hrs
